# Insights into the sustainability of liquid chromatographic methods for favipiravir bioanalysis: a comparative study†

**DOI:** 10.1039/d4ra03017f

**Published:** 2024-06-18

**Authors:** Ahmed Mostafa

**Affiliations:** a Department of Pharmaceutical Chemistry, College of Clinical Pharmacy, Imam Abdulrahman Bin Faisal University King Faisal Road, P.O. Box 1982 Dammam 31441 Eastern Province Saudi Arabia ammostafa@iau.edu.sa ammostaf@uwaterloo.ca (+966) 56 262 3776

## Abstract

The introduction of favipiravir as a broad-spectrum antiviral agent, particularly in treating influenza and exploring its potential against COVID-19, emphasizes the necessity for efficient analytical methods. Liquid chromatography has emerged as a commonly utilized technique for quantifying favipiravir in biological fluids. However, the environmental and health concerns linked to classical analytical methods mean a transition toward green analytical chemistry is required. This study investigates the environmental impact of 19 liquid chromatographic methods utilized in the bioanalysis of favipiravir. Recognizing the importance of eco-friendly practices in pharmaceutical analysis, the study employs three widely accepted greenness assessment tools: Analytical Eco-Scale (AES), Green Analytical Procedure Index (GAPI), and Analytical Greenness Calculator (AGREE). Moreover, it incorporates a comprehensive evaluation on a global scale utilizing the whiteness assessment tool Red-Green-Blue 12 (RGB 12). The comprehensive evaluation aims to extend beyond traditional validation criteria and considerations of green chemistry, providing insights into the development of practically efficient, eco-friendly and economical analytical methods for favipiravir determination. This study emphasizes the necessity of planning for the environmental impact and overall sustainability of analytical methods before laboratory trials. Additionally, the integration of greenness/whiteness evaluation in method validation protocols is strongly advocated, emphasizing the importance of critical and global evaluations in analytical chemistry.

## Abstract 2

The introduction of favipiravir as a broad-spectrum antiviral agent, particularly in treating influenza and exploring its potential against COVID-19, emphasizes the necessity for efficient analytical methods.

## Graphical abstract

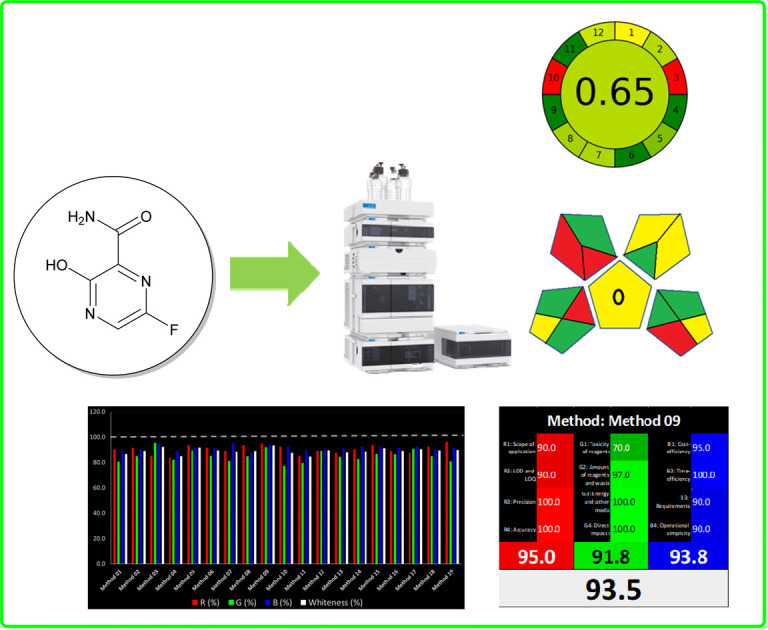

## Introduction

**Fig. 1:**
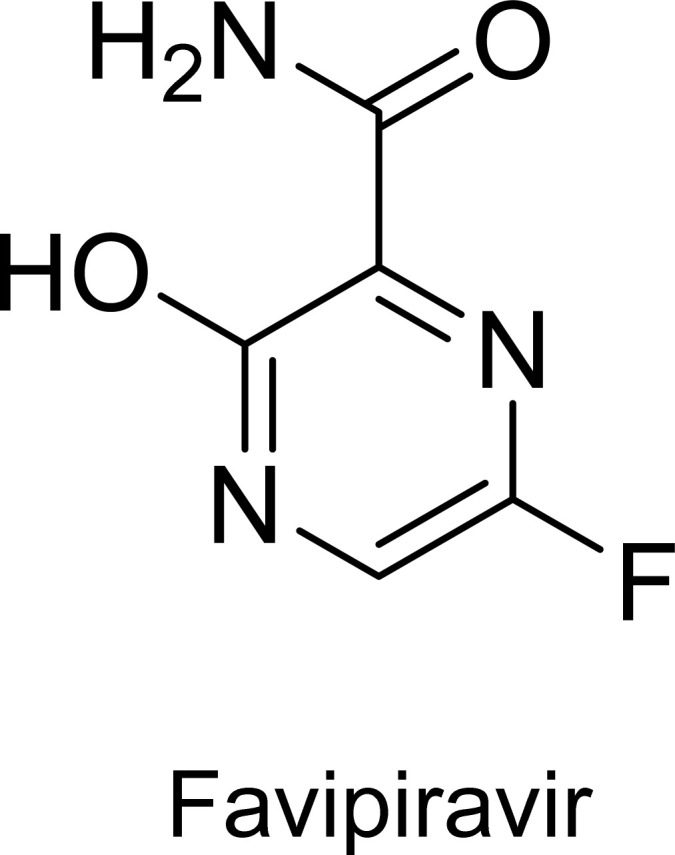
Chemical structure of the antiviral agent favipiravir.

Favipiravir (6-fluoro-3-hydroxypyrazine-2-carboxamide), with the molecular formula C_5_H_4_FN_3_O_2_ (Fig. 1), is a hydrophobic molecule with low water solubility (8.7 mg mL^−1^) and a short half-life.^1^ This results in its rapid clearance from the body through the kidneys in its hydroxylated form.^1^ It is a broad-spectrum antiviral agent utilized for the treatment of various strains of influenza viruses. Its mechanism of action involves inhibiting the RNA-dependent RNA polymerase, thus decreasing viral RNA synthesis.^2^ The drug was first approved for use in Japan in 2014 and has since been used for the treatment of influenza in several countries.^3^ More recently, favipiravir has been studied as a potential treatment for COVID-19.^4^

In recent years, the need for sustainable analytical methods for favipiravir analysis has become increasingly critical for its precise and reliable quantification in biological fluids. Traditional liquid chromatographic techniques, while effective, often involve the use of hazardous chemicals and generate considerable waste, posing environmental and health risks.^5^ The adoption of green analytical chemistry principles is essential to mitigate these impacts, ensuring that bioanalytical methods are not only efficient and accurate but also environmentally friendly.^6^ This necessity drives the development and evaluation of sustainable analytical methods for favipiravir bioanalysis, aiming to align with global sustainability goals and promote safer laboratory practices. Several analytical techniques have been utilized to analyze favipiravir, including spectrophotometry,^7–9^ spectrofluorimetry,^10–12^ high performance liquid chromatography – ultraviolet detector (HPLC-UV),^13–16^ capillary electrophoresis,^17^ liquid chromatography – tandem mass spectrometry (LC-MS/MS)^18–22^ and high performance thin liquid chromatography (HPTLC).^16^ These methods have been applied to pharmaceutical dosage forms^7,8,11^ and biological fluids such as plasma,^13–16,18,19,21,23–30^ serum,^20,31–33^ and milk.^29^

Green Analytical Chemistry (GAC) has gained attention since 2000, with the goal of establishing safe analytical methodologies for both individuals and the environment.^34^ The basic principles and recommended practices of GAC aim to strike a balance between effective analyses and safety measures.^22,35^ These fundamental guidelines of GAC have been widely accepted and published.^6^ However, there is a lack of standardized tools and procedures to evaluate how ‘green’ an analytical method is ref. 36. These tools help determine if a specific analytical method is environmentally friendly. It is crucial to compare and integrate such evaluation tools as a standard practice during development and validation of novel eco-friendly analytical methods.^35^ Hence, evaluation tools like the National Environmental Method Index (NEMI),^36^ Analytical Eco-Scale (AES),^37^ Green Analytical Procedure Index (GAPI),^38^ and Analytical Greenness metric (AGREE)^39^ are commonly employed in recent research to assess the eco-friendliness of methods.^22,40–47^ They are regarded as crucial for the development and validation of new environmentally sustainable analytical techniques.

Achieving a balance between the eco-friendliness of analytical techniques and their practical effectiveness poses a significant challenge. The utility of a method is determined by several parameters such as analytical efficiency (accuracy, precision, sensitivity), practical and economic considerations (cost, speed, simplicity), and environmental impact. There is a diversity of opinions on how to prioritize greenness in method selection. Some supporters of GAC advocate for prioritizing greenness, while others may consider it only when other parameters are satisfactory. Moreover, the shift towards more environmentally friendly methods necessitates training for operators, and the accessibility of these sustainable approaches can be affected by economic and social factors.^48^

In the context of this complexity, Sustainable Development (SD) emerges as a multidimensional concept, emphasizing the delicate balance between method effectiveness, practicality and environmental care. The assessment of method sustainability is complicated, and existing greenness assessment tools like NEMI, AES, GAPI, and AGREE may not comprehensively capture all relevant sustainability criteria.^48^ The suggestion of pre-selecting methods based on other parameters before assessing greenness raises questions about fairness and transparency. Another approach involves employing algorithms for comprehensive assessments of analytical methods on a global scale, such as the Red-Green-Blue (RGB) model.^48–50^ This model enables expanding the principles of green chemistry to incorporate other primary colors, with red symbolizing analytical efficiency, green representing ecological and safety considerations, and blue denoting productivity and economic viability. In this RGB model, white signifies an ideal method. Consequently, the concept of White Analytical Chemistry (WAC) emerged, consisting of 12 principles similar to the established guidelines of GAC.^48^ In conclusion, striking a balance between environmental sustainability and practicality in analytical methods requires careful consideration and comprehensive evaluation. Nowak *et al.* introduced a simplified version of the RGB algorithm, called RGB 12 integrated with WAC.^48^ This algorithm enables a rapid assessment of methods against the 12 WAC principles, providing a measure of the method's sustainability or “whiteness”. Several publications used the developed model to assess the whiteness profile of their methods.^17,30,44,51^

It is the responsibility of researchers to evaluate the environmental sustainability and efficiency of newly developed analytical methods. They must consider factors such as environmental impact, human safety, and overall method performance and practicality when choosing the most eco-friendly approach. It is advisable to incorporate clear planning and integrate environmental considerations into method validation protocols to mitigate environmental risks and enhance method efficiency and economic viability. Utilizing various assessment tools can offer a comprehensive understanding of a method's ecological impact.^43,52^

The objective of this study was to assess the ecological impact of 19 liquid chromatographic methods utilized for the bioanalysis of favipiravir. The evaluation was based on the three widely applied greenness assessment tools: AES, GAPI, and AGREE. Moreover, the study conducted a comprehensive evaluation of all 19 methods using the whiteness assessment tool (RGB 12) after establishing fair rules for scoring all 12 evaluated whiteness criteria to minimize subjectivity. Subsequently, the results were compared with those obtained from the greenness assessment tools. This comprehensive approach aimed to provide an evaluation of methods beyond validation criteria and green chemistry considerations. This study offers valuable insights into the development of more effective, environmentally friendly, economical, and practically efficient analytical methods for the chromatographic bioanalysis of favipiravir.

## Methodology

All methods reported for the analysis of favipiravir starting from 2012 to 2023 were collected. To ensure an unbiased environmental evaluation, the collected research was classified according to the sample matrix and the separation technique utilized. The main criteria for selecting methods were: (1) utilization of chromatography: the method must employ chromatographic techniques for analysis and (2) application for detecting favipiravir in biological matrices: the method must be specifically applied to the detection of favipiravir in biological matrices (*e.g.*, plasma, serum, urine). Consequently, chromatographic methods used for favipiravir analysis in pharmaceutical formulations were excluded because they did not meet the second criterion of the inclusion criteria, which focused on detecting favipiravir in biological matrices. Methods for pharmaceutical formulations typically involve simpler sample preparation procedures, such as the dilute and shoot approach, which are not suitable for biological matrices. Biological samples require more complex preparation techniques to handle the presence of proteins, lipids, and other interfering substances, necessitating a separate evaluation to ensure consistency and fairness in our comparative study. Subsequently, the environmental impact and efficiency profiles of these methods were evaluated utilizing three widely recognized assessment metrics: the AES,^37^ GAPI,^38^ and AGREE,^39^ along with the whiteness metric of RGB 12.^48^ Below is a summary of the tools employed in this study.

## Greenness and whiteness assessment tools

### Analytical eco-scale (AES)

AES assesses the environmental safety of analytical methods, assigning a total score out of 100, starting at 100 for the greenest level with no penalty points (PPs).^37^ PPs are calculated based on various parameters like reagent usage, occupational hazards, waste generation and energy consumption, impacting the overall score. A score above 75 is considered green, 50–75 is acceptable, and below 50 is inadequately green. This method provides comprehensive information on health, safety and ecological hazards compared to the NEMI tool. For further details, readers are referred to ref. 37.

### Green analytical procedure index (GAPI)

This metric, developed by Plotka-Wasylka, was initially introduced in 2019.^38^ It provides a reliable and comprehensive environmental evaluation of the complete analytical methodology, spanning from sample collection to final analysis. This tool covers sample preparation, solvents and reagents, instrumentation and an extra quantification mark. Using a color-coded pictogram, GAPI classifies the environmental safety of each step, with green indicating a safe procedure and red highlighting non-ecofriendly operations. Fig. S1 in file 1 of the ESI† shows a description of the GAPI pictograms. For further details, readers are referred to ref. 38.

### Analytical greenness metric (AGREE)

This metric, introduced by Pereira *et al.* in 2020, is a simple and automated software designed for assessing the greenness of methods.^39^ The AGREE pictogram consists of 12 sections, each aligning with the principles of GAC. These sections, including the central zone, are color-coded from red to green. The color is determined by the method's greenness score, a fraction ranging from zero to one.^39^ This score is automatically calculated and displayed in the middle zone. The AGREE tool is freely available online through https://mostwiedzy.pl. A description of AGREE pictograms is provided in Fig. S2 in file 1 of the ESI.†

### Red-green-blue 12 model (RGB 12)

This model, proposed by Paweł Nowak and Paweł Koscielniak in 2019,^49^ utilizes three colors to signify important attributes: red denotes analytical performance, green signifies adherence to the principles of green chemistry, and blue indicates practical effectiveness. The resulting color is ascertained by combining these primary colors, with their intensities represented as the Color Score (CS). The CS is calculated on a scale from 0 to 100%. These calculations can be performed utilizing a readily accessible Excel spreadsheet.^48^

### Application of the greenness and whiteness evaluation metrics to the methods used for the bioanalysis of favipiravir

The summarized bioanalytical methods utilized for favipiravir analysis using chromatography are presented in Table 1. The AES tool provided digital results without figures. Table 2 shows AES calculations for each method. GAPI uses colored pictograms (green, yellow, and red) to assess the greenness of a method, where green indicates the most eco-friendly approach, while red signifies one that is environmentally harmful. The calculated scores for the methods reported using the GAPI tool are presented in Table 3. Likewise, the AGREE pictogram features three colors similar to the GAPI pictogram, albeit with differing degrees of saturation that gradually intensify according to digital assessments of importance. The comprehensive results for AGREE are centrally displayed within each pictogram. Fig. S3 in file 1 of the ESI† illustrates the calculations for the reported methods utilizing the AGREE metric. Summary results of the greenness assessment of the methods reported using AES, GAPI and AGREE metrics are presented in Table 2.

**Table 1:** Summary of the chromatographic methods used for the analysis of favipiravir in biological samples

No.	Extraction technique	Matrix	Sample volume (mL)	Extraction time (min)	Analytes	Analytical technique	Stationary phase	Mobile phase	Column temperature	Flow rate	HPLC run time (min)	Mode of elution	Waste generated (mL)	Ref.
1	Fabric phase extraction (FPSE)	Human plasma and breast milk	0.5	60	Favipiravir	HPLC-UV	C18 (ODS) shim-pack column (150 mm × 4.6 mm × 5 μm)	Acetonitrile-10 mmol L^−1^ orthophosphoric acid (25 : 75, v/v)	30	0.8	5	Isocratic	4.0	29
2	Protein precipitation	Human serum and plasma	0.25	11	Favipiravir	HPLC-MS/MS	Reverse-phase phenomenex C18 column (50 mm × 4.6 mm, 5 μm)	0.1% formic acid in water – 0.1% formic acid in MOH	25	1	4	Gradient	4.0	32
3	Protein precipitation	Human plasma	1	10.5	Favipiravir and meropenem	UPLC-UV	BEH C18	Acetonitrile-potassium dihydrogen phosphate (pH 3) (10 : 90)	NA	0.3	4.3	Isocratic	1.3	16
4	Volumetric absorptive microsampling (VAMS)	Whole blood	NA	51	Favipiravir	HPLC-UV	C18 column (waters, Sunfire™ 5 μm; 250 × 4.6 mm)	Acetonitrile-0.2% formic acid-20 mmol L^−1^ sodium dihydrogen phosphate pH 3.5	30	0.8	12	Gradient	9.6	33
5	Protein precipitation	Mouse and human plasma	0.1	15–17	Favipiravir	HPLC-MS/MS	Kinetex® F5 column 2.1 × 100 mm 2.6 μm (phenomenex)	0.1% acetic acid and methanol with 0.1% acetic acid	NA	0.6	3	Gradient	1.8	23
6	Menthol-assisted LLME	Human plasma	1.5	25	Favipiravir	HPLC-UV	Thermo® Hypersil ODS C18 column (150 mm × 4.6 mm, 5 μm)	50 mmol L^−1^ phosphate buffer (pH = 2.5) and acetonitrile in a ratio of 60 : 40, v/v	30	1	7	Isocratic	7.0	15
7	Protein precipitation	Human plasma	0.2	15	Favipiravir	HPLC-UV	Phenomenex Kinetex®, C18 (150 × 4.6 mm, 5 μm)	0.1% formic acid in water with 0.08% aqueous ammonia, 0.1% formic acid in acetonitrile with 0.08% aqueous ammonia	40	1	9	Gradient	9.0	28
8	QuEChERS	Rat plasma	0.05	60	Favipiravir, hydroxychloroquine, oseltamivir, and remdesivir	UPLC-MS/MS	Shim-pack GISS C18 column (150 mm × 2.1 mm, 1.9 μm)	Water and acetonitrile, both with 0.1% (v/v) formic acid	45	0.25	13	Gradient	3.25	18
9	Protein precipitation	Human plasma	1	11	Favipiravir, remdesivir and dexamethasone	UPLC-UV	Reversed phase BEH C18 (150 mm × 2.1 mm, 1.7 μm)	Methanol, acetonitrile and water acidified with orthophosphoric acid (pH 4) as a mobile phase in the following ratio: (15 : 35 : 50, by volume)	25	0.3	4.33	Isocratic	1.3	27
10	A gadolinium-based magnetic ionic liquid for supramolecular dispersive liquid–liquid microextraction (DLLME)	Human plasma	2–3 mL	12	Favipiravir	HPLC-UV	Thermo® Hypersil ODS C18 column (150 × 4.6 mm, 5 μm)	50 mmol L^−1^ phosphate buffer (pH = 2.5) and acetonitrile in a 60 : 40, v/v	30	1	5	Isocratic	5	13
11	Liquid–liquid extraction (LLE)	Spiked human plasma	1	40	Favipiravir	HPLC-UV	Symmetry® C18-(250 mm 4.6 mm, 5 μm particle size)	Methanol/acetonitrile:20 mmol L^−1^ phosphate buffer (pH 3.1) 30 : 10 : 60, v/v/v	NA	1	12	Isocratic	12	25
12	Protein precipitation	Human plasma	0.5	9	Favipiravir	HPLC-MS/MS	Eclipse plus C18column (50 × 4.6 mm, 3.5 μm)	Methanol-0.2% acetic acid (20 : 80, v/v)	25	0.6	3	Isocratic	1.8	21
13	Protein precipitation	Human plasma	0.2	6	Favipiravir	UPLC-MS/MS	Acquity UPLC® HSS C18 (100 × 2.1 mm, 1.8 μm)	10 mmol L^−1^ ammonium formate + 0.1% formic acid: methanol	25	0.35	4.5	Gradient	1.6	26
14	Protein precipitation then online solid phase extraction (SPE)	Human serum	0.05	5	Favipiravir, remdesivir and its active metabolite, chloroquine, hydroxychloroquine, lopinavir, ritonavir, and azithromycin	2D-HPLC-MS/MS	MassTox^®^ TDM MasterColumn^®^ series A (chromsystems)	Loading pump: phase A1 water and B1 acetonitrile-formic acid (99.9 : 0.01, v/v) eluting pump: phase A2 MassTox^®^ TDM series A mobile phase 1 and phase B2 MassTox^®^ TDM series A mobile phase 2 (chromsystems)	NA	0.6	5	Gradient	3 + 6 for online SPE	20
15	Homogeneous liquid–liquid microextraction (LLME)	Human plasma	1.5	13	Favipiravir	HPLC-UV	Hypersil ODS C18 column (150 mm × 4.6 mm, 5 μm)	60% of aqueous phosphate buffer (50 mmol L^−1^, pH was adjusted to 2.5): 40% acetonitrile	30	1	5	Isocratic	5	14
16	Protein precipitation	Human plasma	0.2	20.5	Favipiravir	UPLC-MS/MS	Acquity UPLC BEH HILIC column (2.1 × 100 mm, 1.7 μm)	Acetonitrile and 0.005% ammonia in water (75 : 25, v/v)	40	0.25	2	Isocratic	0.5	19
17	SPE	Human serum	0.15	4	Favipiravir	HPLC-UV	Chromolith high resolution RP-column (100 × 4.6 mm)	0.1% phosphoric acid/acetonitrile (95 : 5)	30	2	3	Isocratic	6	31
18	LLE	Spiked human plasma	2	40	Favipiravir	HPLC-UV	Chromasil C18 (250 × 4.6 mm × 5 μm)	Methanol/water, 35 : 65, pH 3	40	0.8	9.39	Isocratic	7.5	24
19	Protein precipitation	Spiked human plasma	1	40	Favipiravir, aspirin, atenolol, atorvastatin, losartan and remdesivir	UPLC-UV	ACQUITY UPLC^®^ BEH C18 (2.1 × 150 mm, and 1.7 μm particle size)`	0.03 mol L^−1^ Brij-35, 0.15 mol L^−1^ sodium dodecyl sulfate, and 0.02 mol L^−1^ sodium dihydrogen phosphate (pH 5.0) as the mobile phase A (90%) and *n*-propanol as the mobile phase B (10%)	30	0.5 (5.5 min) 0.8 (2.5 min)	8	Isocratic	4.25	30

**Table 2:** Comparison of the greenness profiles of the reported methods determining favipiravir in biological samples using Analytical Eco-Scale (AES), Green Analytical Procedure Index (GAPI) and Analytical GREEnness (AGREE) metric

Method	Analytical eco-scale		GAPI	AGREE	Ref.
1	**Reagents**	PPs^a^	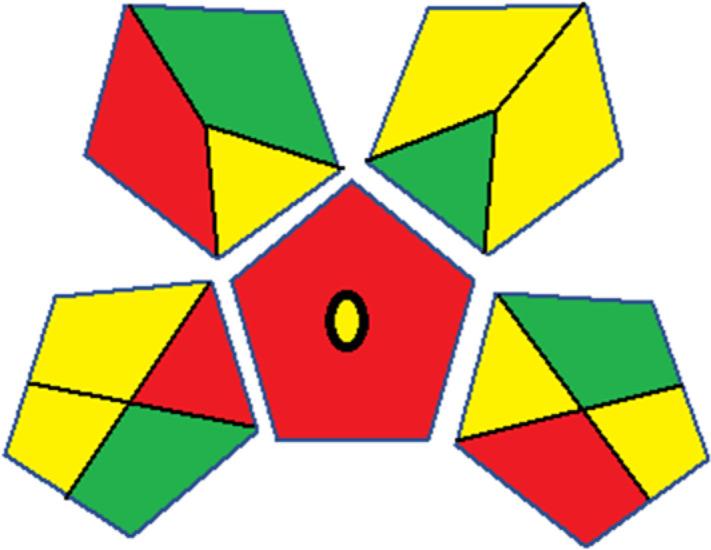	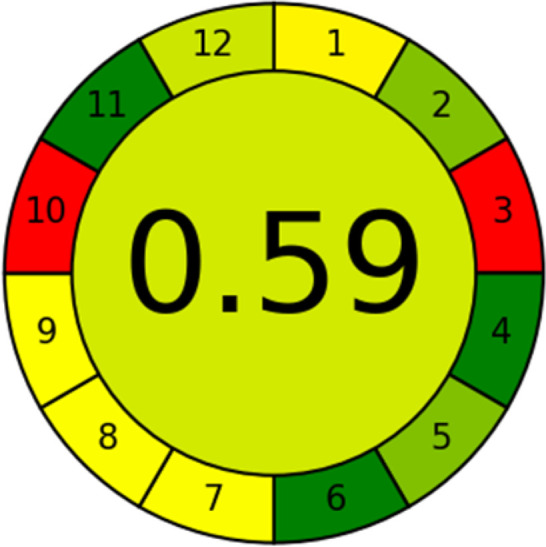	29
	Polycaprolactone-*block*-polydimethylsiloxane-*block*-polycaprolactone polymer	0			
	Methyl trimethoxysilane	2			
	Acetone	4			
	Methylene chloride	2			
	Trifluoroacetic acid	4			
	Methanol	6			
	Acetonitrile	4			
	Phosphoric acid	2			
	**Instruments**				
	Vortex	0			
	Centrifuge	0			
	Conditioning device (50 *C* × *h*)	2			
	Ultrasonic bath	2			
	Magnetic stirring	0			
	HPLC-UV	1			
	**Occupational hazard**	0			
	**Waste**	0			
	**Total PPs**	**29**			
	**Eco-scale**	**71**			

2	**Reagents**	PPs	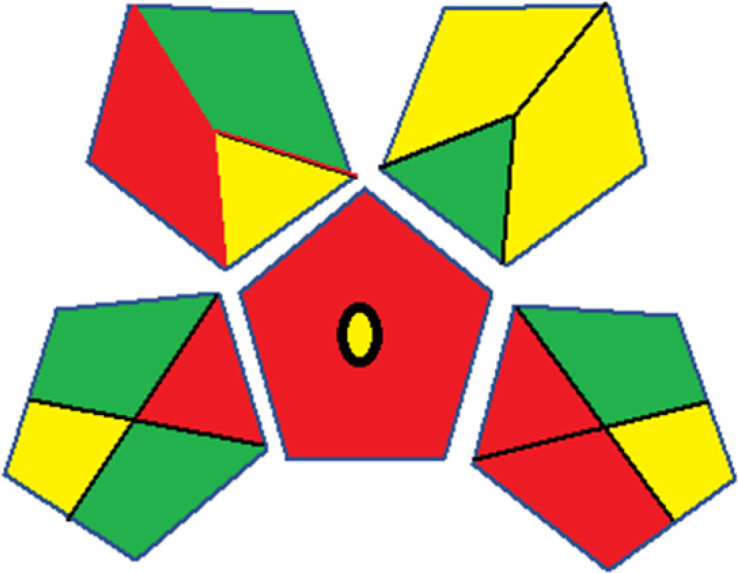	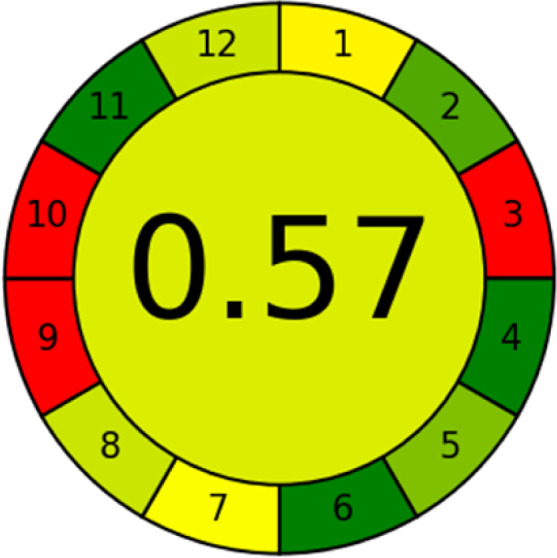	32
	Acetonitrile	4			
	Formic acid	6			
	Methanol	6			
	**Instruments**				
	Vortex	0			
	Centrifuge	0			
	LC-MS/MS	2			
	**Occupational hazard**	0			
	**Waste**	3			
	**Total PPs**	**21**			
	**Eco-scale**	**79**			

3	**Reagents**	PPs	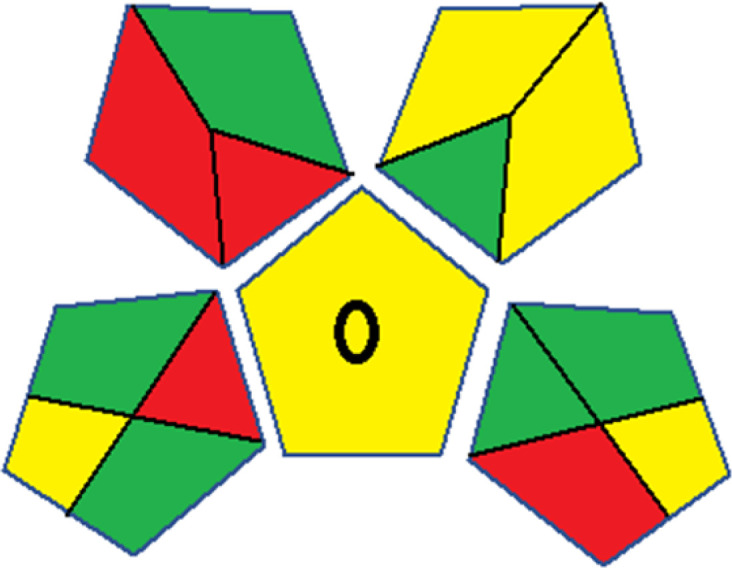	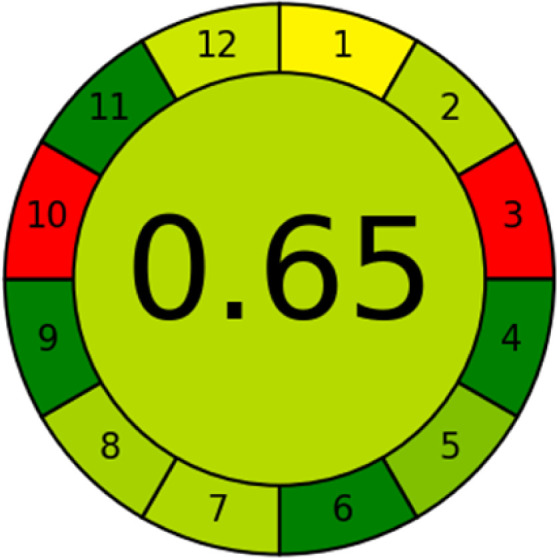	16
	Acetonitrile	4			
	Phosphoric acid	2			
	Potassium dihydrogen orthophosphate	0			
	**Instruments**				
	Vortex	0			
	Centrifuge	0			
	UHPLC	0			
	**Occupational hazard**	0			
	**Waste**	3			
	**Total PPs**	**9**			
	**Eco-scale**	**91**			

4	**Reagents**	PPs	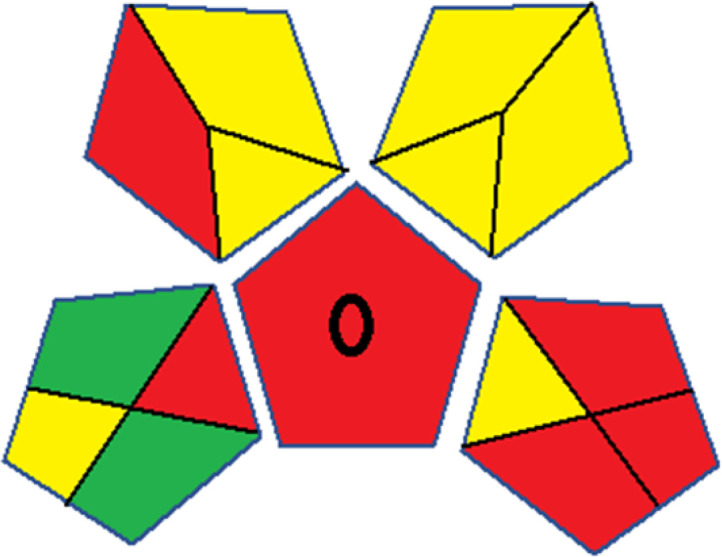	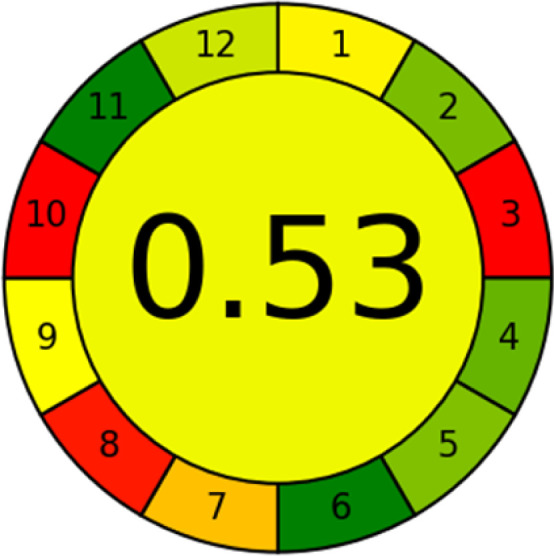	33
	Acetonitrile	4			
	Formic acid	6			
	Sodium dihydrogen phosphate	0			
	Methanol	6			
	**Instruments**				
	Vortex	0			
	Centrifuge	0			
	Ultrasonic bath	0			
	HPLC-DAD	1			
	Solvent evaporator	2			
	**Occupational hazard**	3			
	**Waste**	5			
	**Total PPs**	**27**			
	**Eco-scale**	**73**			

5	**Reagents**	PPs	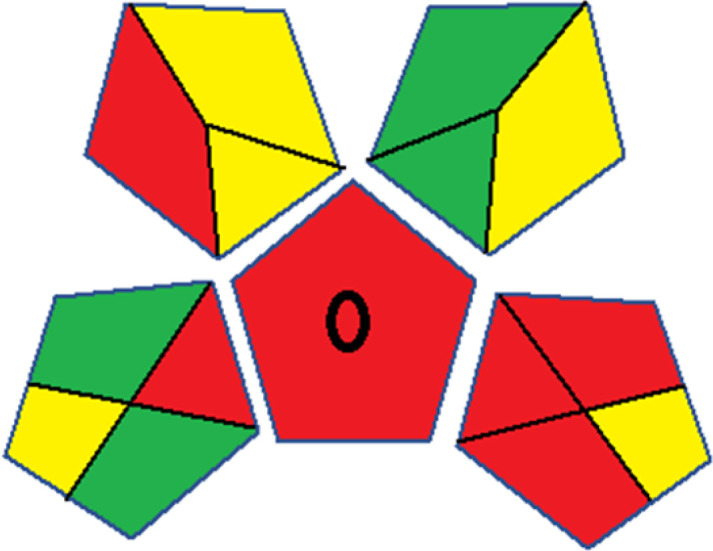	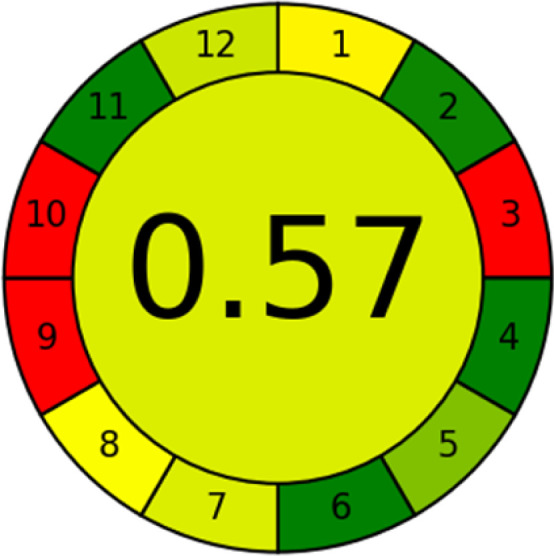	23
	Methanol	6			
	Acetic acid	4			
	**Instruments**				
	Vortex	0			
	Centrifuge	0			
	Solvent evaporator	2			
	LC-MS/MS	2			
	**Occupational hazard**	3			
	**Waste**	3			
	**Total PPs**	**20**			
	**Eco-scale**	**80**			

6	**Reagents**	PPs	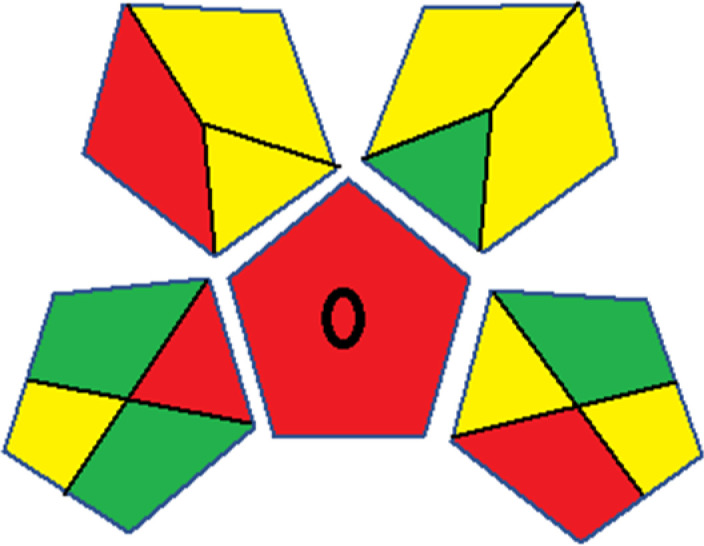	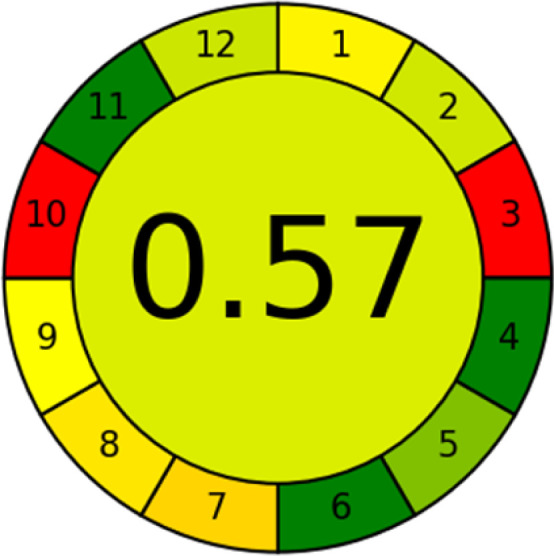	15
	Tetrahydrofuran	6			
	Menthol	1			
	Trichloroacetic acid	4			
	Acetonitrile	4			
	Potassium dihydrogen phosphate	0			
	Phosphoric acid	2			
	**Instruments**				
	Vortex	0			
	Centrifuge	0			
	HPLC-UV	1			
	**Occupational hazard**	0			
	**Waste**	3			
	**Total PPs**	**21**			
	**Eco-scale**	**79**			

7	**Reagents**	PPs	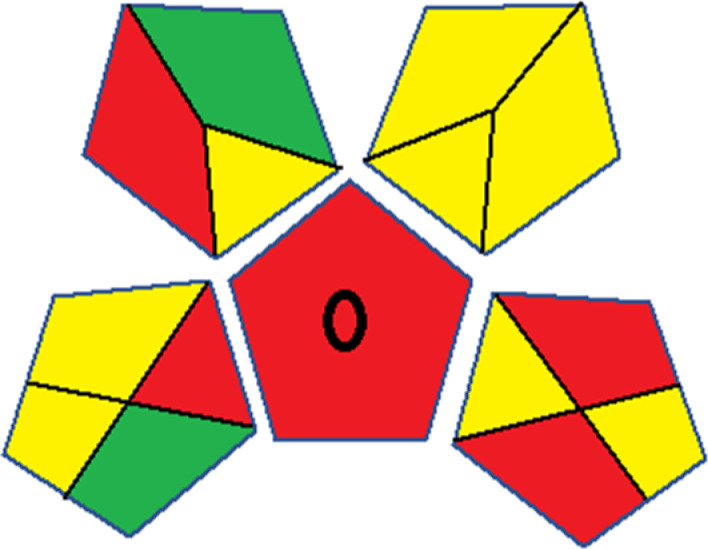	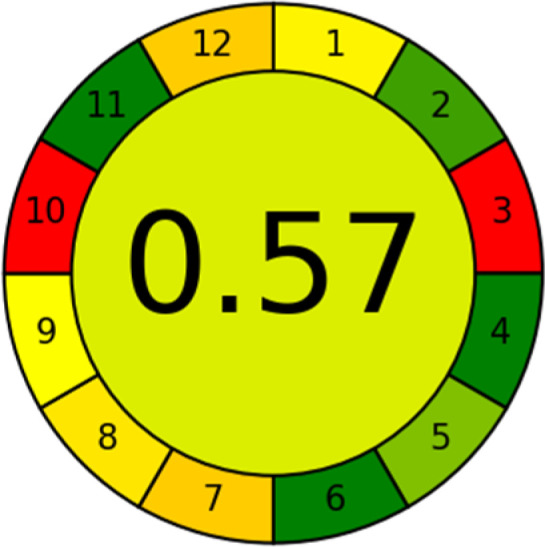	28
	Acetonitrile	4			
	Formic acid	6			
	Ammonia	6			
	Methanol	6			
	**Instruments**				
	Vortex	0			
	Centrifuge	0			
	HPLC-UV	1			
	**Occupational hazard**	3			
	**Waste**	3			
	**Total PPs**	**29**			
	**Eco-scale**	**71**			

8	**Reagents**	PPs	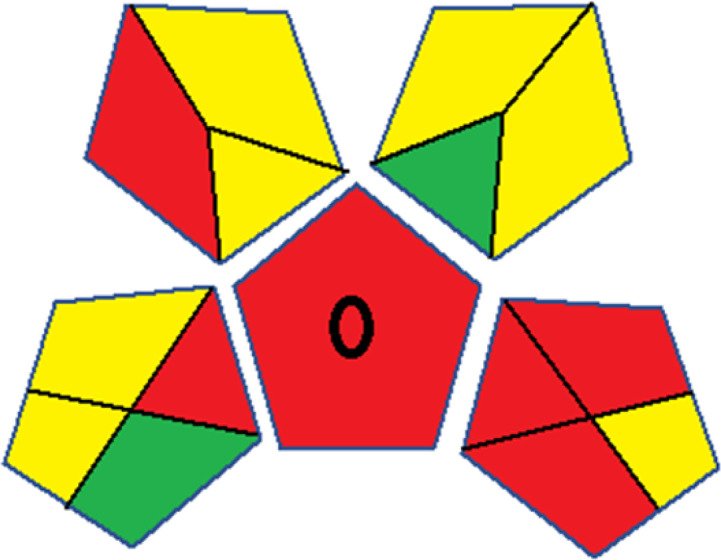	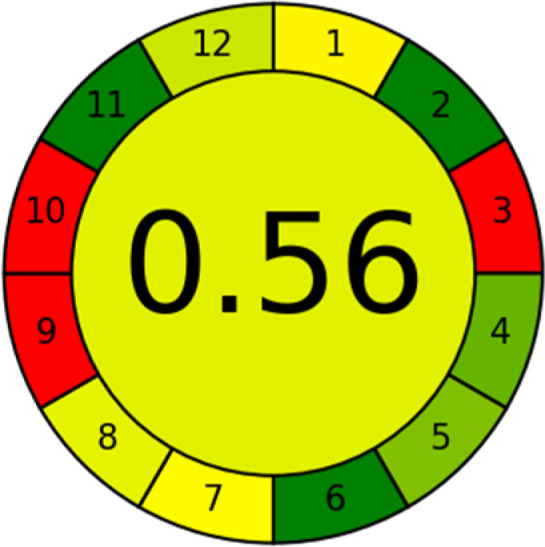	18
	Acetonitrile	4			
	Formic acid	6			
	NaCl	0			
	Anhydrous magnesium sulphate	0			
	Methanol	6			
	**Instruments**				
	Vortex	0			
	Centrifuge	0			
	UHPLC-MS/MS	2			
	Solvent evaporator	2			
	**Occupational hazard**	3			
	**Waste**	3			
	**Total PPs**	**26**			
	**Eco-scale**	**74**			

9	**Reagents**	PPs	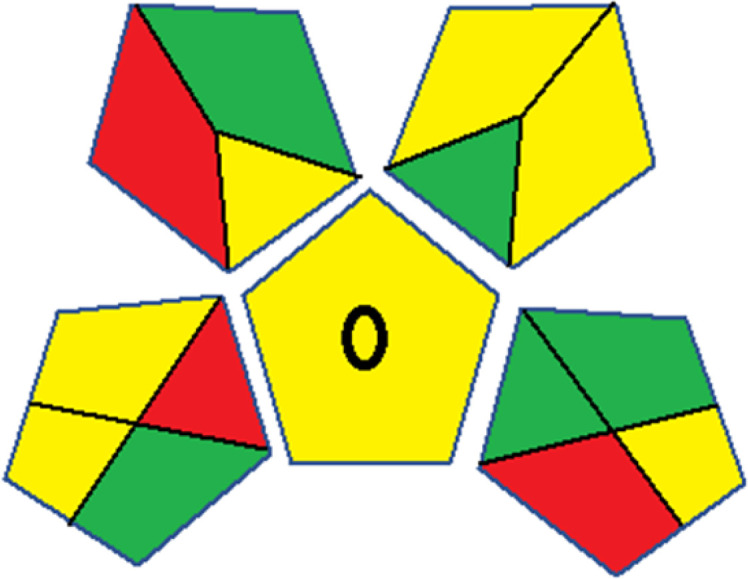	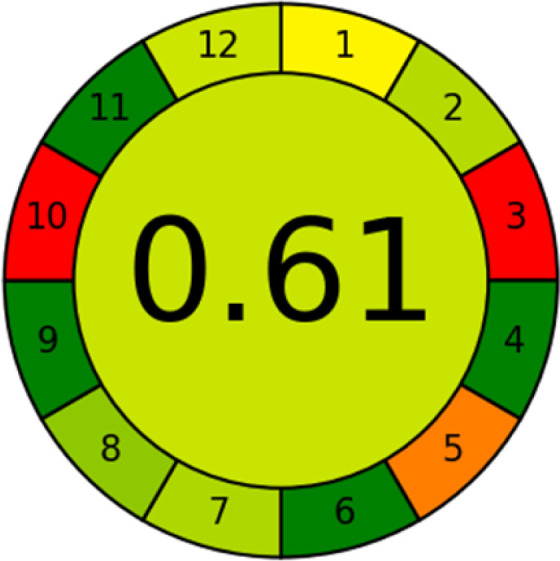	27
	Acetonitrile	4			
	Phosphoric acid	2			
	Methanol	6			
	**Instruments**				
	Vortex	0			
	Centrifuge	0			
	UHPLC-UV	0			
	**Occupational hazard**	0			
	**Waste**	3			
	**Total PPs**	**15**			
	**Eco-scale**	**85**			

10	**Reagents**	PPs	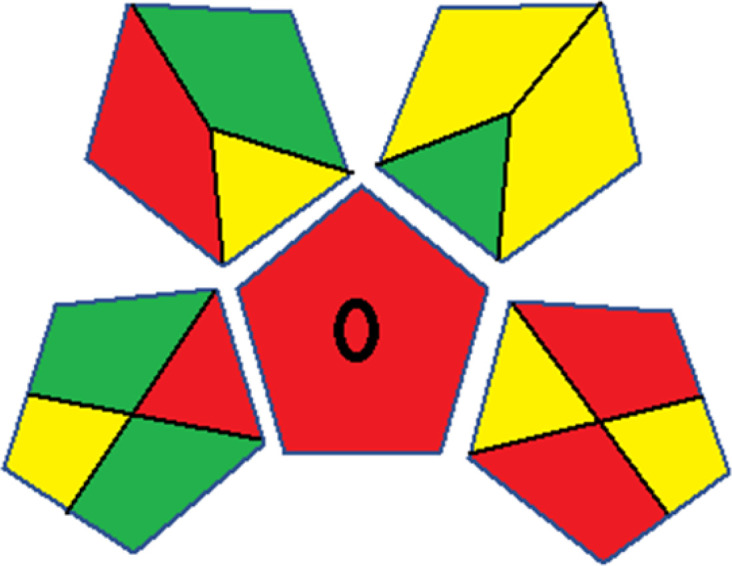	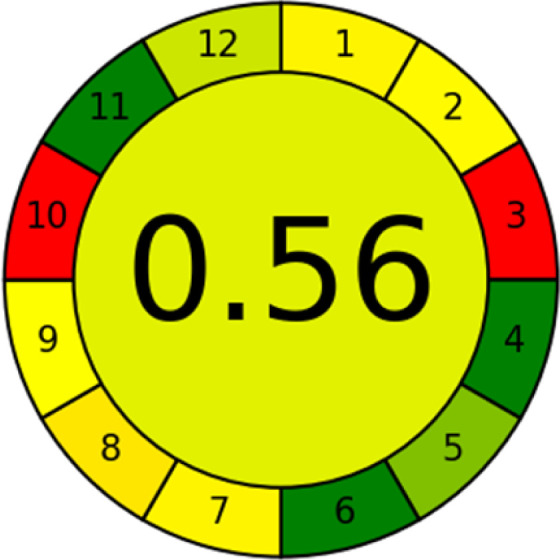	13
	Acetonitrile	4			
	Phosphoric acid	2			
	Gadolinium(iii) chloride hexahydrate	1			
	Trihexyl (tetradecyl)phosphonium chloride	4			
	Dichloromethane	4			
	Perchloric acid	4			
	Tetrahydrofuran	6			
	**Instruments**				
	Reflux	2			
	Vacuum evaporator (drying)	2			
	Vortex	0			
	Centrifuge	0			
	HPLC-UV	1			
	**Occupational hazard**	0			
	**Waste**	3			
	**Total PPs**	**33**			
	**Eco-scale**	**67**			

11	**Reagents**	PPs	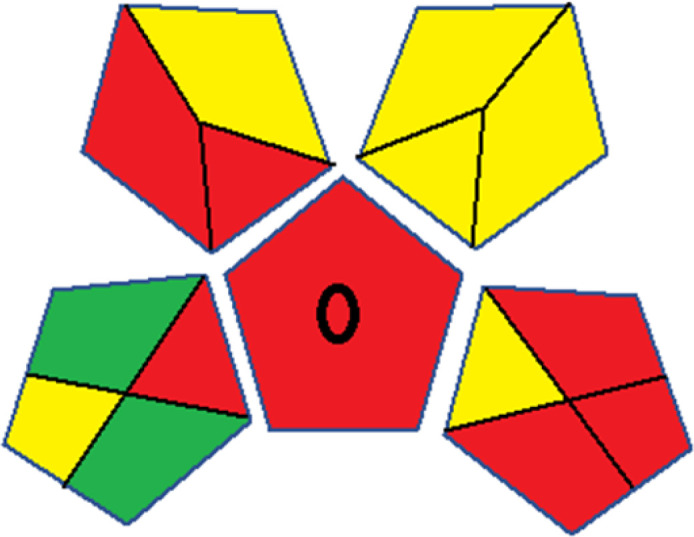	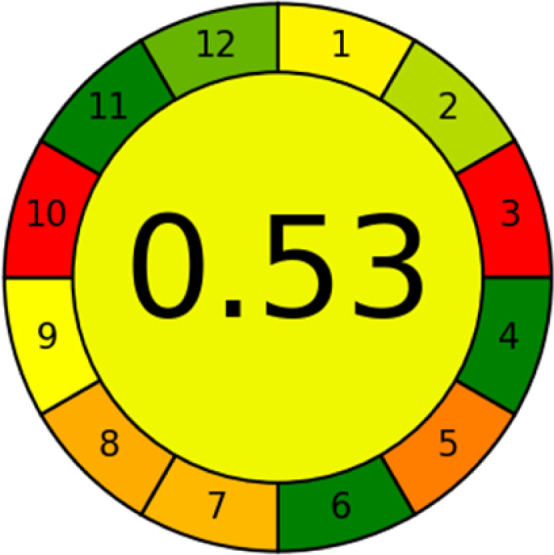	25
	Acetonitrile	4			
	Methanol	6			
	Phosphate buffer	0			
	Dichloromethane	4			
	**Instruments**				
	Solvent evaporator	2			
	Vortex	0			
	Centrifuge	0			
	HPLC-UV	1			
	**Occupational hazard**	3			
	**Waste**	5			
	**Total PPs**	**25**			
	**Eco-scale**	**75**			

12	**Reagents**	PPs	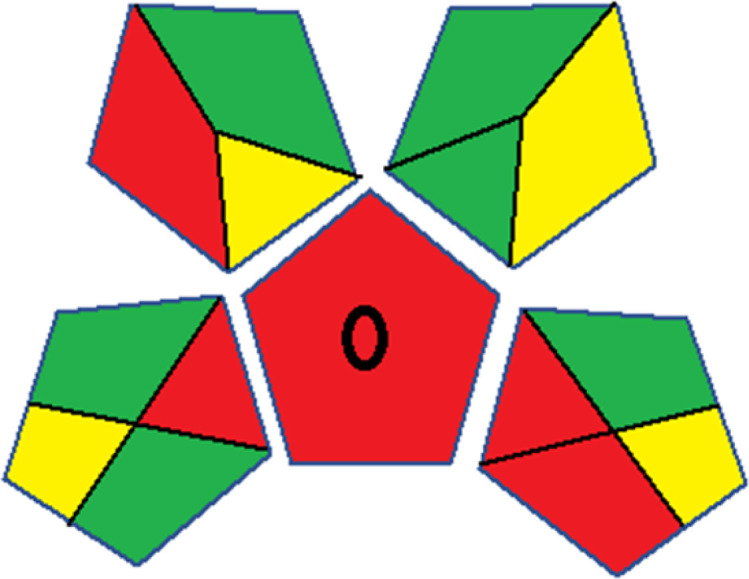	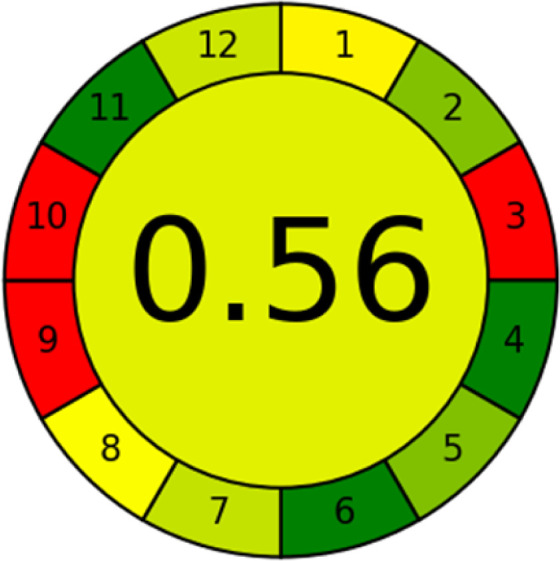	21
	Methanol	6			
	Acetic acid	4			
	**Instruments**				
	Vortex	0			
	Centrifuge	0			
	LC-MS/MS	2			
	**Occupational hazard**	0			
	**Waste**	3			
	**Total PPs**	**15**			
	**Eco-scale**	**85**			

13	**Reagents**	PPs	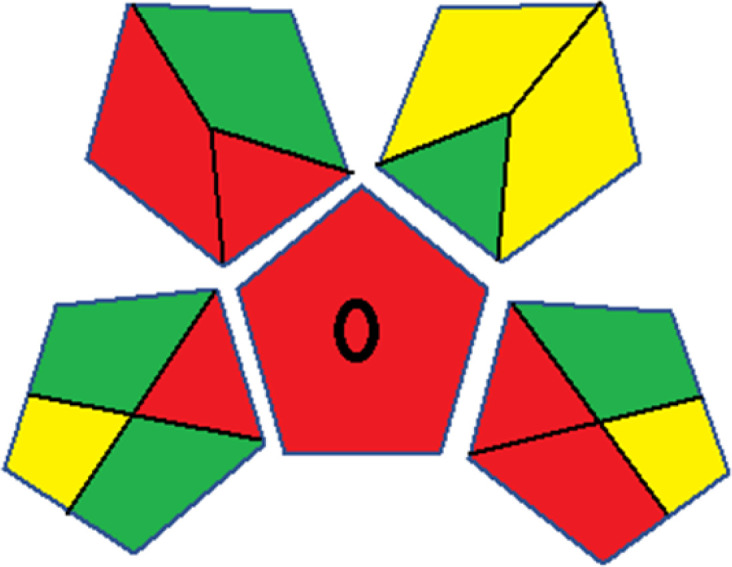	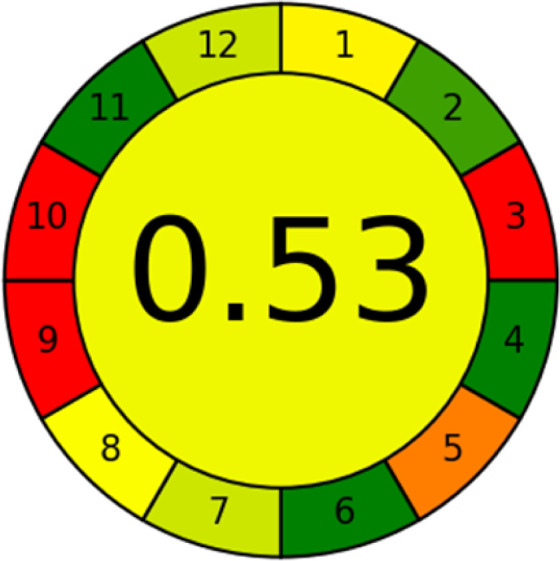	25
	Methanol	6			
	Formic acid	6			
	Ammonium format	1			
	Acetonitrile	4			
	**Instruments**				
	Vortex	0			
	Centrifuge	0			
	LC-MS/MS	2			
	**Occupational hazard**	0			
	**Waste**	3			
	**Total PPs**	**22**			
	**Eco-scale**	**78**			

14	**Reagents**	PPs	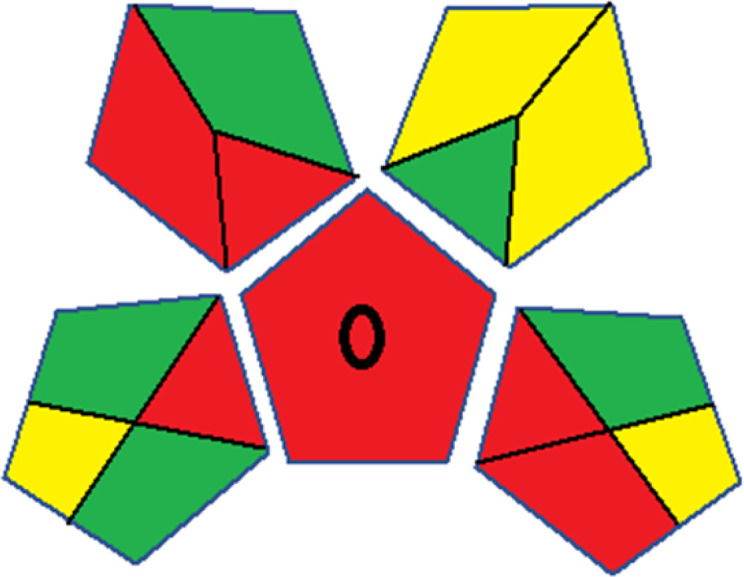	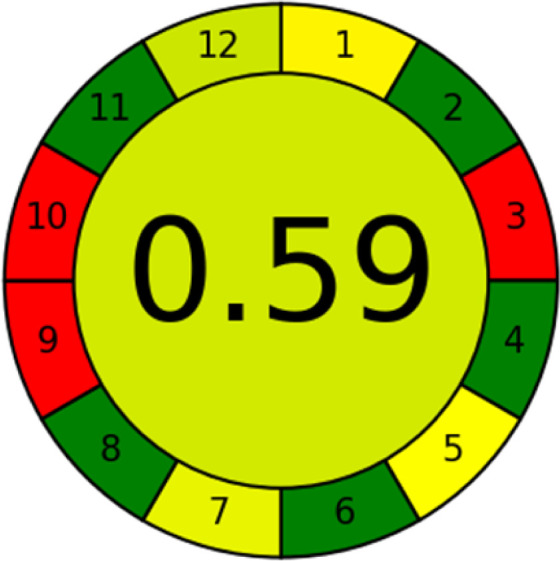	20
	Acetonitrile	4			
	Formic acid	6			
	Methanol	6			
	**Instruments**				
	Vortex	0			
	Centrifuge	0			
	LC-MS/MS	2			
	**Occupational hazard**	0			
	**Waste**	3			
	**Total PPs**	**21**			
	**Eco-scale**	**79**			

15	**Reagents**	PPs	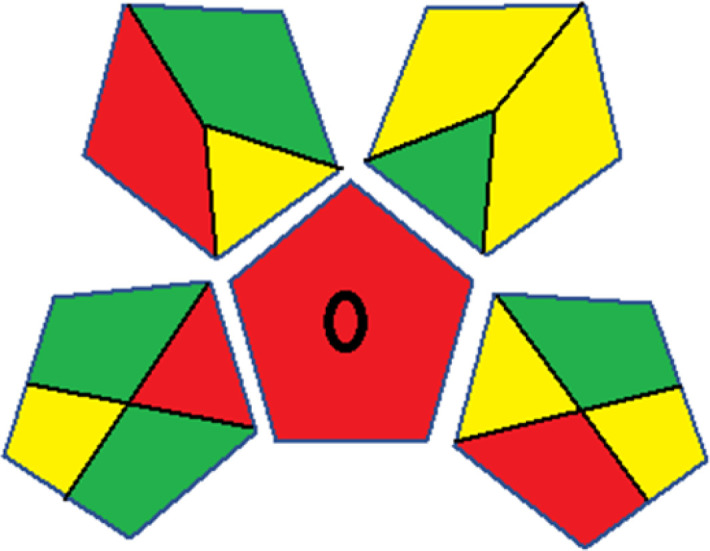	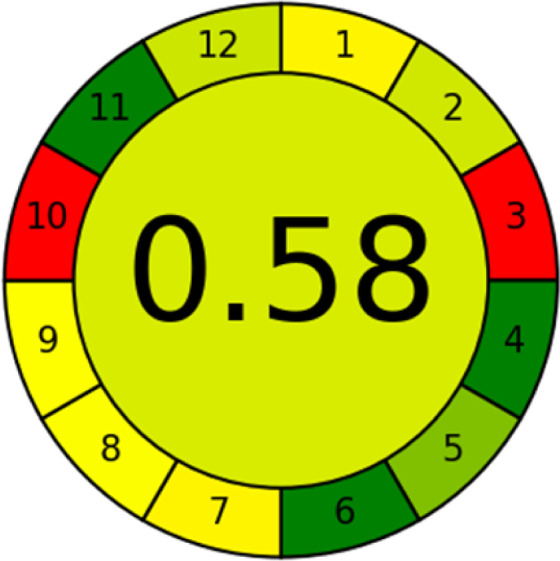	14
	Acetonitrile	4			
	Phosphoric acid	2			
	Perchloric acid	4			
	Tetrahydrofuran	6			
	**Instruments**				
	Vortex	0			
	Centrifuge	0			
	HPLC-UV	1			
	**Occupational hazard**	0			
	**Waste**	3			
	**Total PPs**	**20**			
	**Eco-scale**	**80**			

16	**Reagents**	PPs	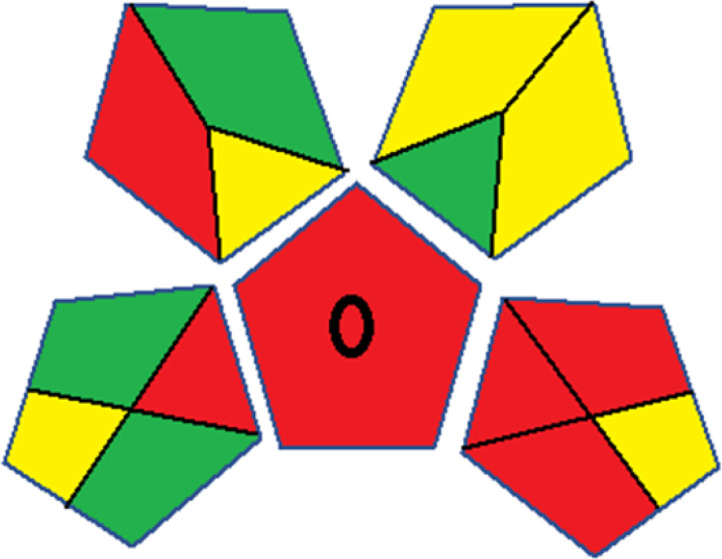	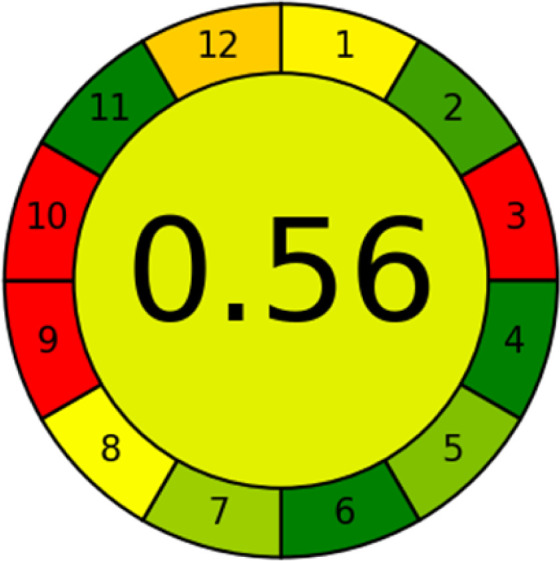	19
	Acetonitrile	4			
	Ammonia	6			
	Methanol	6			
	**Instruments**				
	Vortex	0			
	Centrifuge	0			
	UHPLC-MS/MS	2			
	**Occupational hazard**	3			
	**Waste**	3			
	**Total PPs**	**24**			
	**Eco-scale**	**76**			

17	**Reagents**	PPs	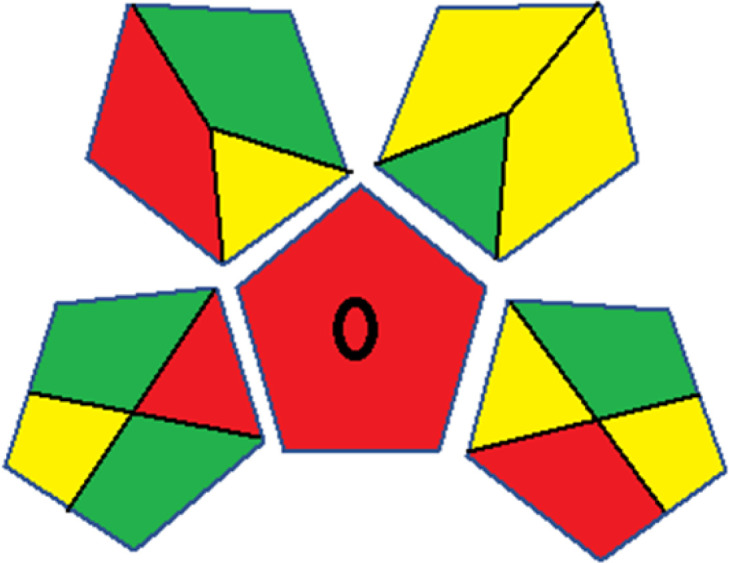	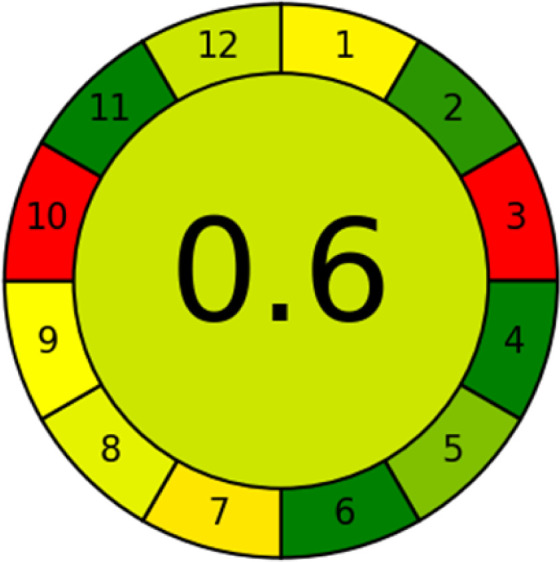	31
	Acetonitrile	4			
	Phosphoric acid	2			
	HCl	4			
	**Instruments**				
	Vortex	0			
	Centrifuge	0			
	HPLC-UV	1			
	**Occupational hazard**	0			
	**Waste**	3			
	**Total PPs**	**14**			
	**Eco-scale**	**86**			

18	**Reagents**	PPs	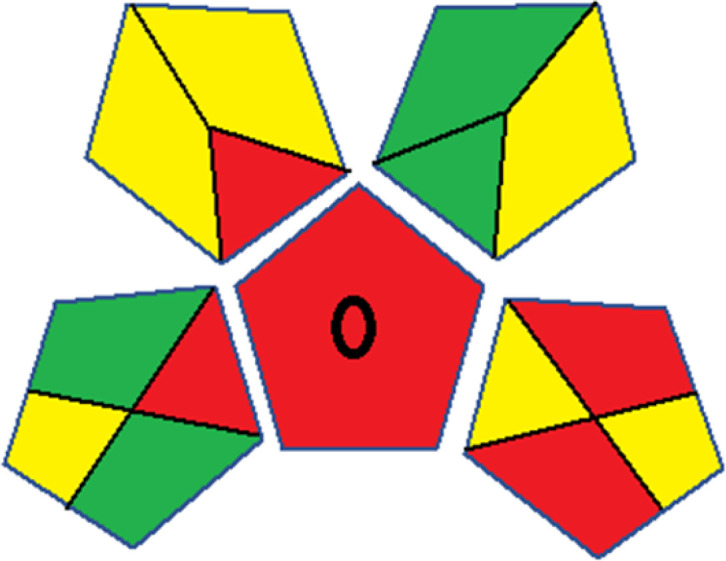	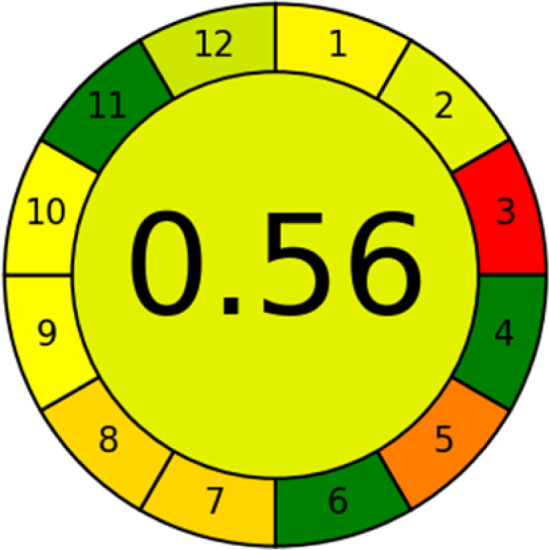	24
	Methanol	6			
	Ethyl acetate	4			
	Phosphoric acid	2			
	**Instruments**				
	Vortex	0			
	Centrifuge	0			
	Solvent evaporator	2			
	HPLC-UV	1			
	**Occupational hazard**	3			
	**Waste**	3			
	**Total PPs**	**21**			
	**Eco-scale**	**79**			

19	**Reagents**	PPs	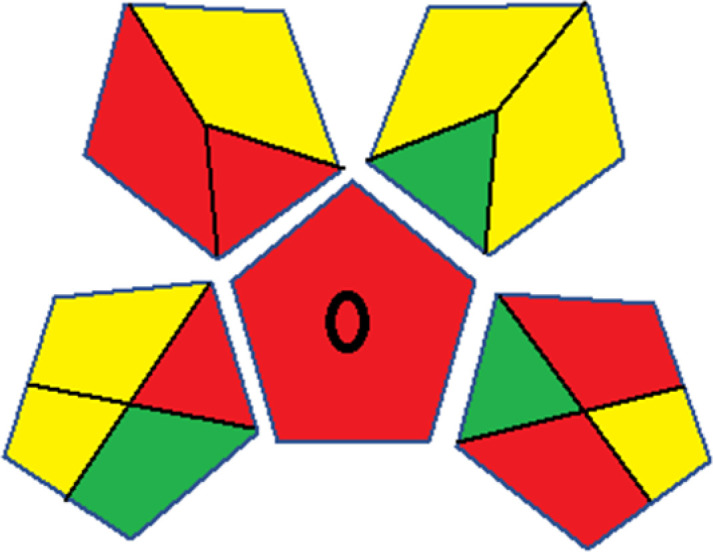	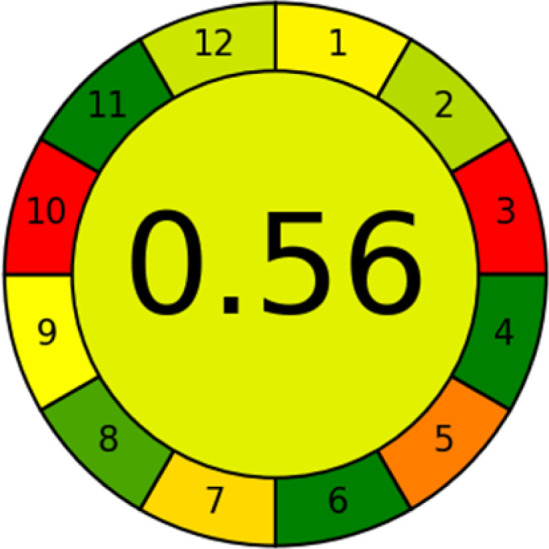	30
	Brij-35	4			
	Methanol	6			
	Sodium dodecyl sulfate	4			
	Sodium dihydrogen phosphate	0			
	Propanol	6			
	**Instruments**				
	Vortex	0			
	Centrifuge	0			
	Solvent evaporator	2			
	UPLC-UV	0			
	**Occupational hazard**	3			
	**Waste**	3			
	**Total PPs**	**28**			
	**Eco-scale**	**72**			

a PPs for analytical eco-scale were calculated as per ref. 37 as follows: (1) reagent amount PPs: <10 mL (g) = 1; 10–100 mL (g) = 2; >100 mL (g) = 3. (2) Reagent hazard PPs: none = 0; less severe = 1; more severe = 2. Then total PPs = amount PPs × hazard PPs. (3) Energy PPs: ≤ 0.1 kWh per sample = 0, ≤ 1.5 kWh per sample = 1, > 1.5 kWh per sample = 2. (4) Occupational hazard PPs: analytical process hermitization = 0; emission of vapors and gases to air = 3. (5) Waste PPs: none = 0; <1 mL (g) = 1; 1–10 mL (g) = 3; >10 mL (g) = 5.

**Table 3:** Detailed calculations of the greenness profiles of the reported methods using the Green Analytical Procedure Index (GAPI)

Method	Category	Assessment	GAPI pictograms	Ref.
1	**Sample preparation**		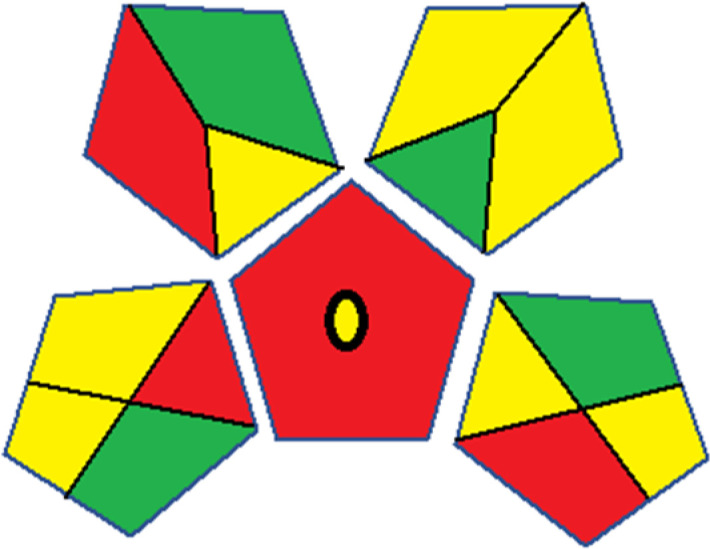	29
	Collection (1)	Offline (red)		
	Preservation (2)	None (green)		
	Transport (3)	Required (yellow)		
	Storage (4)	Under normal conditions (yellow)		
	Type of method: direct or indirect (5)	Extraction required (red)		
	Scale of extraction (6)	Micro-extraction (yellow)		
	Solvents/reagents used (7)	Non-green solvents/reagents used (red)		
	Additional treatments (8)	None (green)		
	**Reagent and solvents**			
	Amount (9)	< 10 mL (green)		
	Health hazard (10)	NFPA = 2, moderate toxicity (yellow)		
	Safety hazard (11)	NFPA = 3, high flammability (yellow)		
	**Instrumentation**			
	Energy (12)	≤ 1.5 kW h per sample (yellow)		
	Occupational hazard (13)	Hermetic sealing of analytical procedure (green)		
	Waste (14)	1–10 mL (yellow)		
	Waste treatment (15)	No treatment (red)		

2	**Sample preparation**		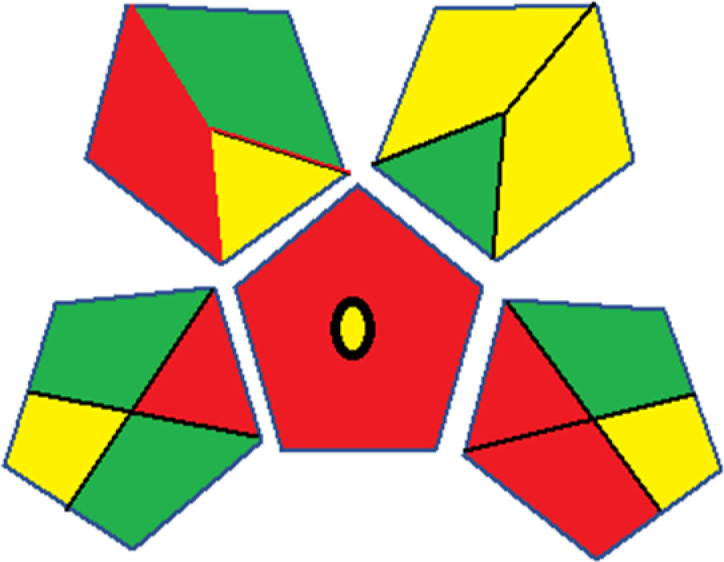	32
	Collection (1)	Offline (red)		
	Preservation (2)	None (green)		
	Transport (3)	Required (yellow)		
	Storage (4)	None (yellow)		
	Type of method: direct or indirect (5)	Extraction required (red)		
	Scale of extraction (6)	Micro-extraction (yellow)		
	Solvents/reagents used (7)	Non-green solvents/reagents used (red)		
	Additional treatments (8)	None (green)		
	**Reagent and solvents**			
	Amount (9)	< 10 mL (green)		
	Health hazard (10)	NFPA = 2, moderate toxicity (yellow)		
	Safety hazard (11)	NFPA = 3, high flammability (yellow)		
	**Instrumentation**			
	Energy (12)	> 1.5 kW h per sample (red)		
	Occupational hazard (13)	Hermetic sealing of analytical procedure (green)		
	Waste (14)	1–10 mL (yellow)		
	Waste treatment (15)	No treatment (red)		

3	**Sample preparation**		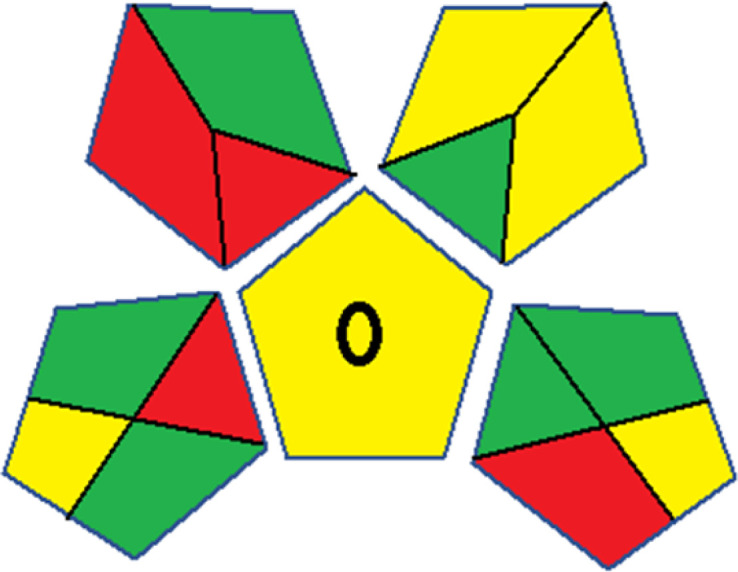	16
	Collection (1)	Offline (red)		
	Preservation (2)	None (green)		
	Transport (3)	Required (yellow)		
	Storage (4)	None (green)		
	Type of method: direct or indirect (5)	Simple treatment (yellow)		
	Scale of extraction (6)	Macro-extraction (red)		
	Solvents/reagents used (7)	Non-green solvents/reagents used (red)		
	Additional treatments (8)	None (green)		
	**Reagent and solvents**			
	Amount (9)	< 10 mL (green)		
	Health hazard (10)	NFPA = 2, moderate toxicity (yellow)		
	Safety hazard (11)	NFPA = 3, high flammability (yellow)		
	**Instrumentation**			
	Energy (12)	≤0.1 kW h per sample (green)		
	Occupational hazard (13)	Hermetic sealing of analytical procedure (green)		
	Waste (14)	1–10 mL (yellow)		
	Waste treatment (15)	No treatment (red)		

4	**Sample preparation**		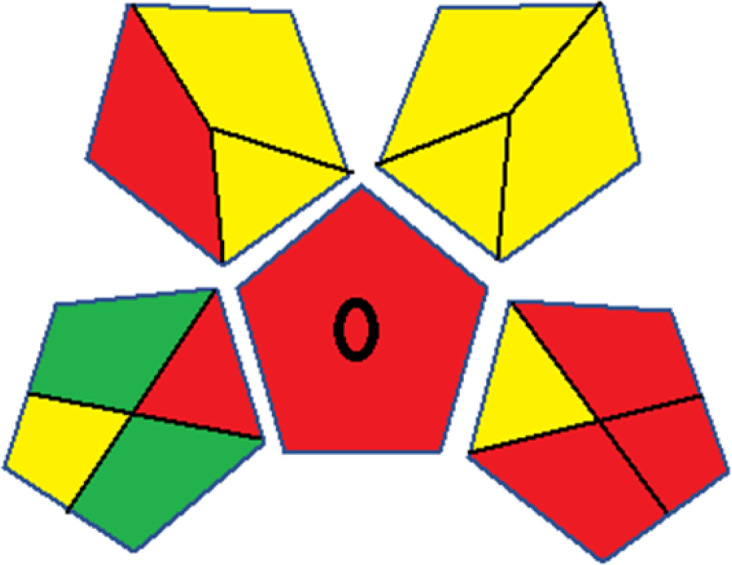	33
	Collection (1)	Offline (red)		
	Preservation (2)	None (green)		
	Transport (3)	Required (yellow)		
	Storage (4)	None (green)		
	Type of method: direct or indirect (5)	Extraction required (red)		
	Scale of extraction (6)	Micro-extraction (yellow)		
	Solvents/reagents used (7)	Non-green solvents/reagents used (red)		
	Additional treatments (8)	Simple treatment (yellow)		
	**Reagent and solvents**			
	Amount (9)	10–100 mL (yellow)		
	Health hazard (10)	NFPA = 2, moderate toxicity (yellow)		
	Safety hazard (11)	NFPA = 3, high flammability (yellow)		
	**Instrumentation**			
	Energy (12)	≤1.5 kW h per sample (yellow)		
	Occupational hazard (13)	Emission of vapours to the atmosphere (red)		
	Waste (14)	>10 mL (red)		
	Waste treatment (15)	No treatment (red)		

5	**Sample preparation**		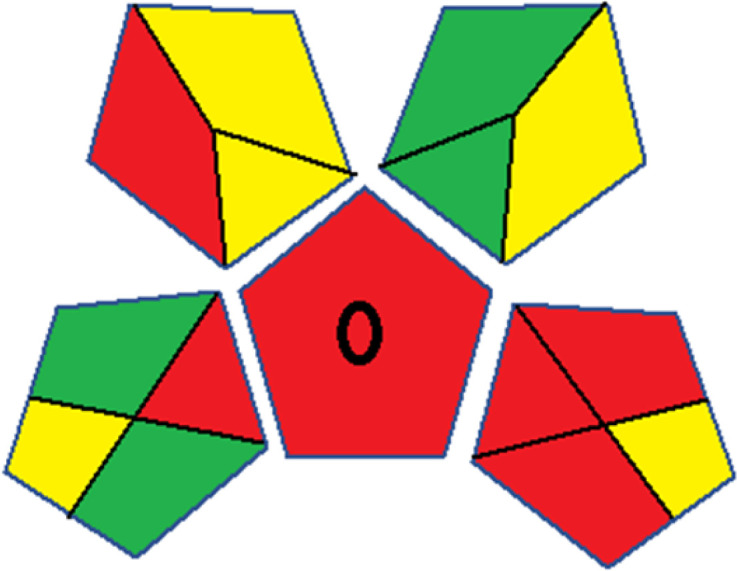	23
	Collection (1)	Offline (red)		
	Preservation (2)	None (green)		
	Transport (3)	Required (yellow)		
	Storage (4)	None (green)		
	Type of method: direct or indirect (5)	Extraction required (red)		
	Scale of extraction (6)	Micro-extraction (yellow)		
	Solvents/reagents used (7)	Non-green solvents/reagents used (red)		
	Additional treatments (8)	Simple treatment (yellow)		
	**Reagent and solvents**			
	Amount (9)	<10 mL (green)		
	Health hazard (10)	NFPA = 1, slight toxicity (green)		
	Safety hazard (11)	NFPA = 3, high flammability (yellow)		
	**Instrumentation**			
	Energy (12)	>1.5 kW h per sample (red)		
	Occupational hazard (13)	Emission of vapours to the atmosphere (red)		
	Waste (14)	1–10 mL (yellow)		
	Waste treatment (15)	No treatment (red)		

6	**Sample preparation**		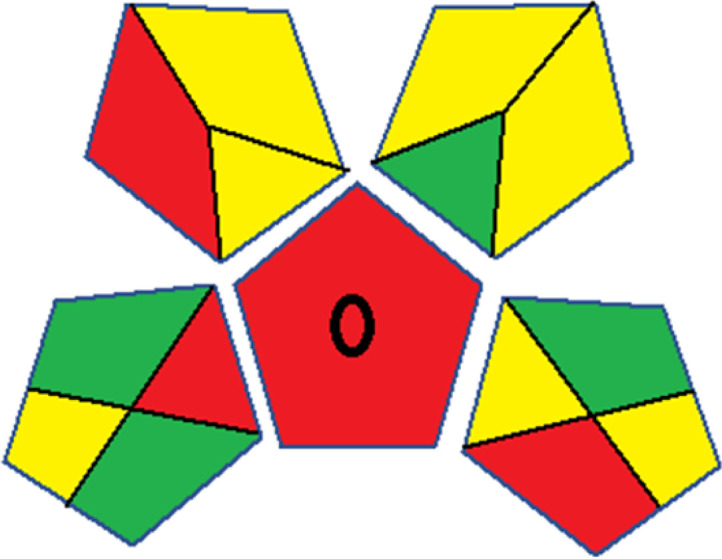	15
	Collection (1)	Offline (red)		
	Preservation (2)	None (green)		
	Transport (3)	Required (yellow)		
	Storage (4)	None (green)		
	Type of method: Direct or indirect (5)	Extraction required (red)		
	Scale of extraction (6)	Micro-extraction (yellow)		
	Solvents/reagents used (7)	Non-green solvents/reagents used (red)		
	Additional treatments (8)	Simple treatment (yellow)		
	**Reagent and solvents**			
	Amount (9)	< 10 mL (green)		
	Health hazard (10)	NFPA = 2, moderate toxicity (yellow)		
	Safety hazard (11)	NFPA = 3, high flammability (yellow)		
	**Instrumentation**			
	Energy (12)	≤1.5 kW h per sample (yellow)		
	Occupational hazard (13)	Hermetic sealing of analytical procedure (green)		
	Waste (14)	1–10 mL (yellow)		
	Waste treatment (15)	No treatment (red)		

7	**Sample preparation**		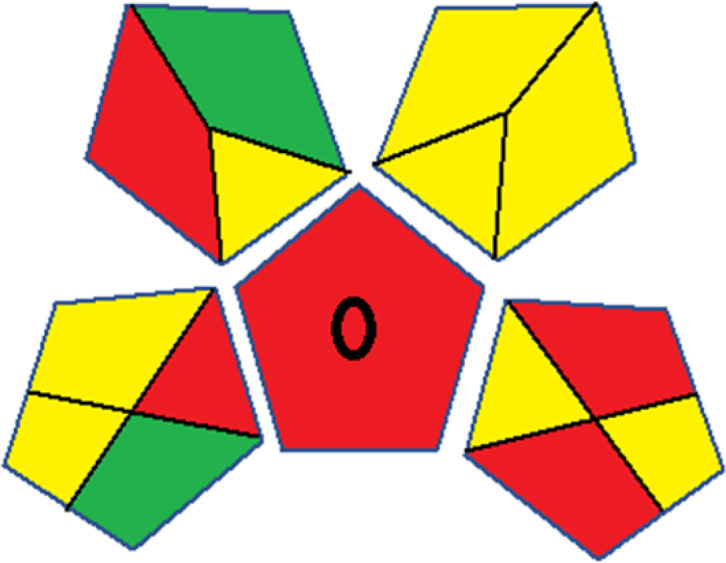	28
	Collection (1)	Offline (red)		
	Preservation (2)	None (green)		
	Transport (3)	Required (yellow)		
	Storage (4)	Under normal conditions (yellow)		
	Type of method: Direct or indirect (5)	Extraction required (red)		
	Scale of extraction (6)	Micro-extraction (yellow)		
	Solvents/reagents used (7)	Non-green solvents/reagents used (red)		
	Additional treatments (8)	None (green)		
	**Reagent and solvents**			
	Amount (9)	10–100 mL (yellow)		
	Health hazard (10)	NFPA = 2, moderate toxicity (yellow)		
	Safety hazard (11)	NFPA = 3, high flammability (yellow)		
	**Instrumentation**			
	Energy (12)	≤1.5 kW h per sample (yellow)		
	Occupational hazard (13)	Emission of vapours to the atmosphere (red)		
	Waste (14)	1–10 mL (yellow)		
	Waste treatment (15)	No treatment (red)		

8	**Sample preparation**		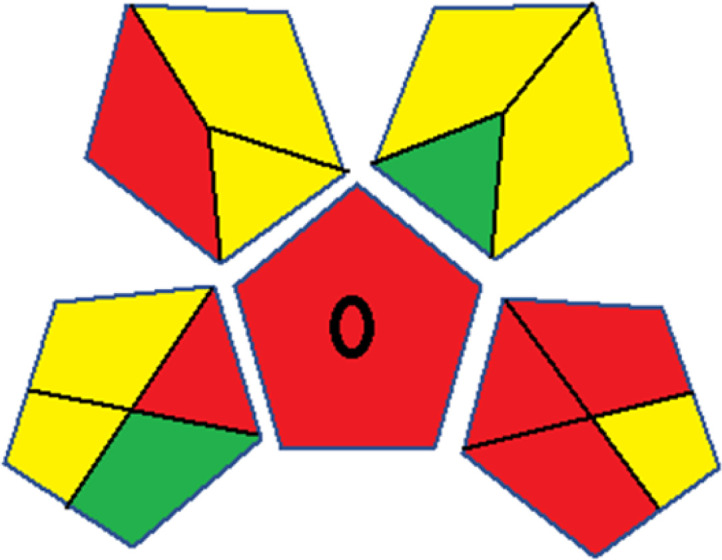	18
	Collection (1)	Offline (red)		
	Preservation (2)	None (green)		
	Transport (3)	Required (yellow)		
	Storage (4)	Under normal conditions (yellow)		
	Type of method: direct or indirect (5)	Extraction required (red)		
	Scale of extraction (6)	Micro-extraction (yellow)		
	Solvents/reagents used (7)	Non-green solvents/reagents used (red)		
	Additional treatments (8)	Simple treatment (yellow)		
	**Reagent and solvents**			
	Amount (9)	< 10 mL (green)		
	Health hazard (10)	NFPA = 2, moderate toxicity (yellow)		
	Safety hazard (11)	NFPA = 3, high flammability (yellow)		
	**Instrumentation**			
	Energy (12)	>1.5 kW h per sample (red)		
	Occupational hazard (13)	Emission of vapours to the atmosphere (red)		
	Waste (14)	1–10 mL (yellow)		
	Waste treatment (15)	No treatment (red)		

9	**Sample preparation**		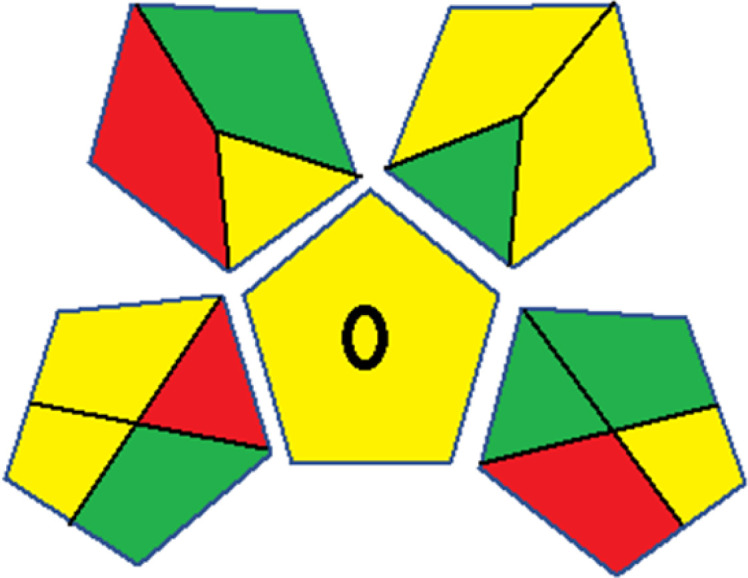	27
	Collection (1)	Offline (red)		
	Preservation (2)	None (green)		
	Transport (3)	Required (yellow)		
	Storage (4)	Under normal conditions (yellow)		
	Type of method: direct or indirect (5)	Simple procedures (yellow)		
	Scale of extraction (6)	Micro-extraction (yellow)		
	Solvents/reagents used (7)	Non-green solvents/reagents used (red)		
	Additional treatments (8)	None (green)		
	**Reagent and solvents**			
	Amount (9)	<10 mL (green)		
	Health hazard (10)	NFPA = 2, moderate toxicity (yellow)		
	Safety hazard (11)	NFPA = 3, high flammability (yellow)		
	**Instrumentation**			
	Energy (12)	≤0.1 kW h per sample (green)		
	Occupational hazard (13)	Hermetic sealing of analytical procedure (green)		
	Waste (14)	1–10 mL (yellow)		
	Waste treatment (15)	No treatment (red)		

10	**Sample preparation**		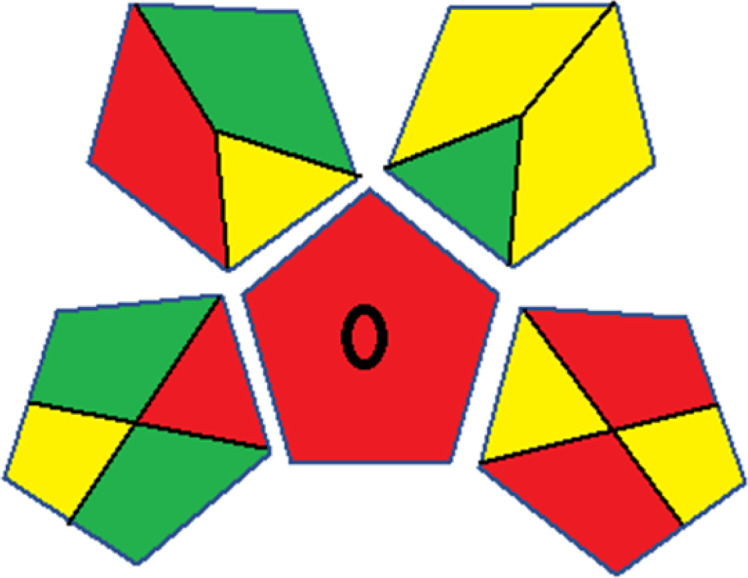	13
	Collection (1)	Offline (red)		
	Preservation (2)	None (green)		
	Transport (3)	Required (yellow)		
	Storage (4)	None (green)		
	Type of method: Direct or indirect (5)	Extraction required (red)		
	Scale of extraction (6)	Micro-extraction (yellow)		
	Solvents/reagents used (7)	Non-green solvents/reagents used (red)		
	Additional treatments (8)	None (green)		
	**Reagent and solvents**			
	Amount (9)	<10 mL (green)		
	Health hazard (10)	NFPA = 2, moderate toxicity (yellow)		
	Safety hazard (11)	NFPA = 3, high flammability (yellow)		
	**Instrumentation**			
	Energy (12)	≤1.5 kW h per sample (yellow)		
	Occupational hazard (13)	Emission of vapours to the atmosphere (red)		
	Waste (14)	1–10 mL (yellow)		
	Waste treatment (15)	No treatment (red)		

11	**Sample preparation**		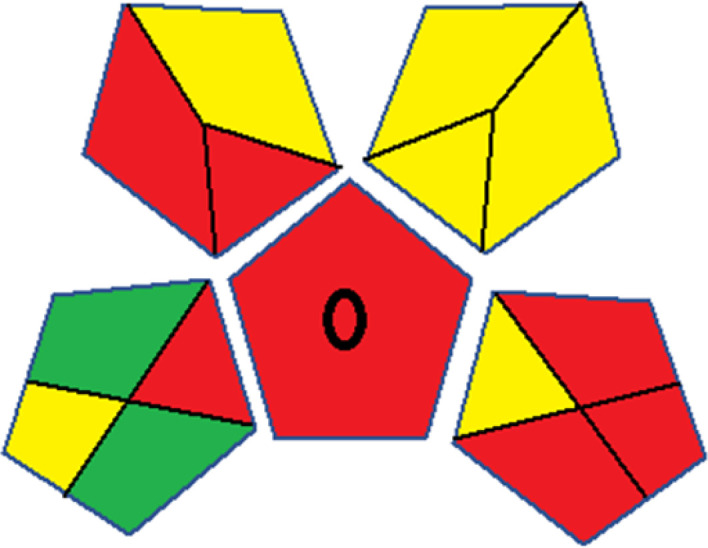	25
	Collection (1)	Offline (red)		
	Preservation (2)	None (green)		
	Transport (3)	Required (yellow)		
	Storage (4)	None (green)		
	Type of method: direct or indirect (5)	Extraction required (red)		
	Scale of extraction (6)	Macro-extraction (red)		
	Solvents/reagents used (7)	Non-green solvents/reagents used (red)		
	Additional treatments (8)	Simple treatments (yellow)		
	**Reagent and solvents**			
	Amount (9)	10–100 mL (yellow)		
	Health hazard (10)	NFPA = 2, moderate toxicity (yellow)		
	Safety hazard (11)	NFPA = 3, high flammability (yellow)		
	**Instrumentation**			
	Energy (12)	≤1.5 kW h per sample (yellow)		
	Occupational hazard (13)	Emission of vapours to the atmosphere (red)		
	Waste (14)	> 10 mL (red)		
	Waste treatment (15)	No treatment (red)		

12	**Sample preparation**		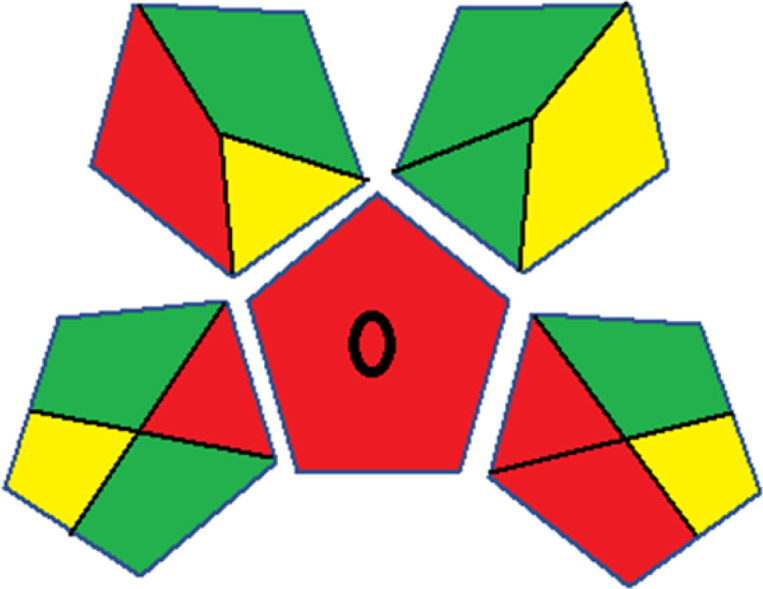	21
	Collection (1)	Offline (red)		
	Preservation (2)	None (green)		
	Transport (3)	Required (yellow)		
	Storage (4)	None (green)		
	Type of method: direct or indirect (5)	Extraction required (red)		
	Scale of extraction (6)	Micro-extraction (yellow)		
	Solvents/reagents used (7)	Non-green solvents/reagents used (red)		
	Additional treatments (8)	None (green)		
	**Reagent and solvents**			
	Amount (9)	< 10 mL (green)		
	Health hazard (10)	NFPA = 1, slight toxicity (green)		
	Safety hazard (11)	NFPA = 3, high flammability (yellow)		
	**Instrumentation**			
	Energy (12)	>1.5 kW h per sample (red)		
	Occupational hazard (13)	Hermetic sealing of analytical procedure (green)		
	Waste (14)	1–10 mL (yellow)		
	Waste treatment (15)	No treatment (red)		

13	**Sample preparation**		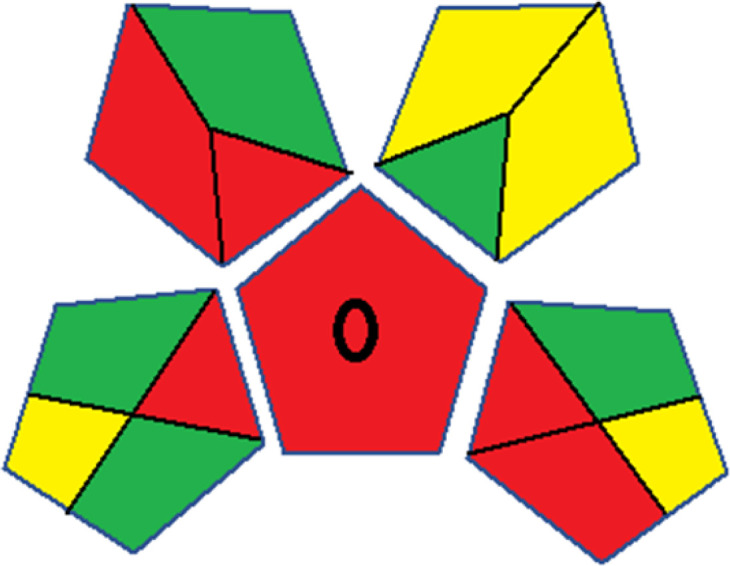	26
	Collection (1)	Offline (red)		
	Preservation (2)	None (green)		
	Transport (3)	Required (yellow)		
	Storage (4)	None (green)		
	Type of method: direct or indirect (5)	Extraction required (red)		
	Scale of extraction (6)	Macro-extraction (red)		
	Solvents/reagents used (7)	Non-green solvents/reagents used (red)		
	Additional treatments (8)	None (green)		
	**Reagent and solvents**			
	Amount (9)	< 10 mL (green)		
	Health hazard (10)	NFPA = 2, moderate toxicity (yellow)		
	Safety hazard (11)	NFPA = 3, high flammability (yellow)		
	**Instrumentation**			
	Energy (12)	>1.5 kW h per sample (red)		
	Occupational hazard (13)	Hermetic sealing of analytical procedure (green)		
	Waste (14)	1–10 mL (yellow)		
	Waste treatment (15)	No treatment (red)		

14	**Sample preparation**		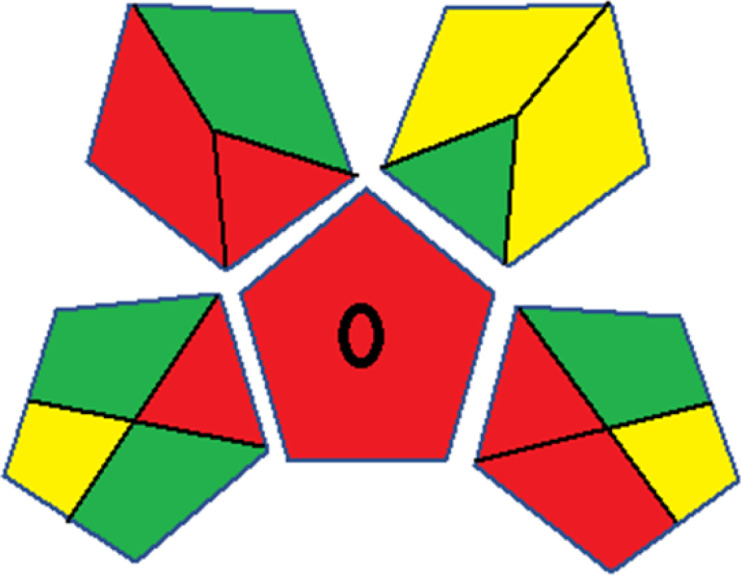	20
	Collection (1)	Offline (red)		
	Preservation (2)	None (green)		
	Transport (3)	Required (yellow)		
	Storage (4)	None (green)		
	Type of method: direct or indirect (5)	Extraction required (red)		
	Scale of extraction (6)	Macro-extraction (red)		
	Solvents/reagents used (7)	Non-green solvents/reagents used (red)		
	Additional treatments (8)	None (green)		
	**Reagent and solvents**			
	Amount (9)	< 10 mL (green)		
	Health hazard (10)	NFPA = 2, moderate toxicity (yellow)		
	Safety hazard (11)	NFPA = 3, high flammability (yellow)		
	**Instrumentation**			
	Energy (12)	>1.5 kW h per sample (red)		
	Occupational hazard (13)	Hermetic sealing of analytical procedure (green)		
	Waste (14)	1–10 mL (yellow)		
	Waste treatment (15)	No treatment (red)		

15	**Sample preparation**		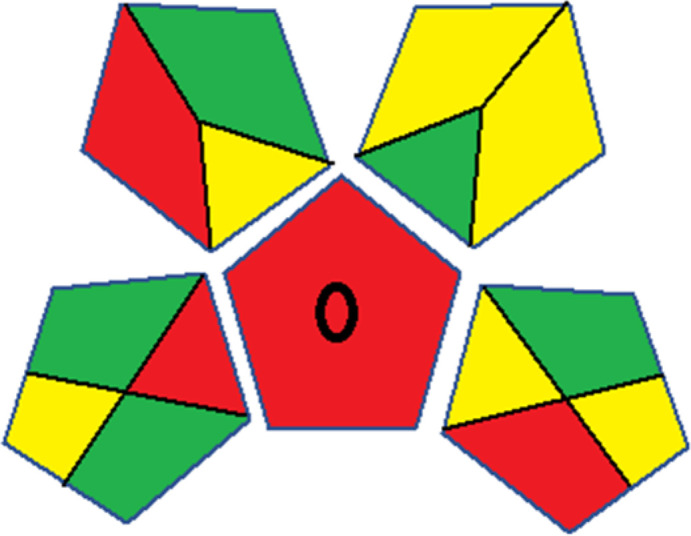	14
	Collection (1)	Offline (red)		
	Preservation (2)	None (green)		
	Transport (3)	Required (yellow)		
	Storage (4)	None (green)		
	Type of method: direct or indirect (5)	Extraction required (red)		
	Scale of extraction (6)	Micro-extraction (yellow)		
	Solvents/reagents used (7)	Non-green solvents/reagents used (red)		
	Additional treatments (8)	None (green)		
	**Reagent and solvents**			
	Amount (9)	< 10 mL (green)		
	Health hazard (10)	NFPA = 2, moderate toxicity (yellow)		
	Safety hazard (11)	NFPA = 3, high flammability (yellow)		
	**Instrumentation**			
	Energy (12)	≤1.5 kW h per sample (yellow)		
	Occupational hazard (13)	Hermetic sealing of analytical procedure (green)		
	Waste (14)	1–10 mL (yellow)		
	Waste treatment (15)	No treatment (red)		

16	**Sample preparation**		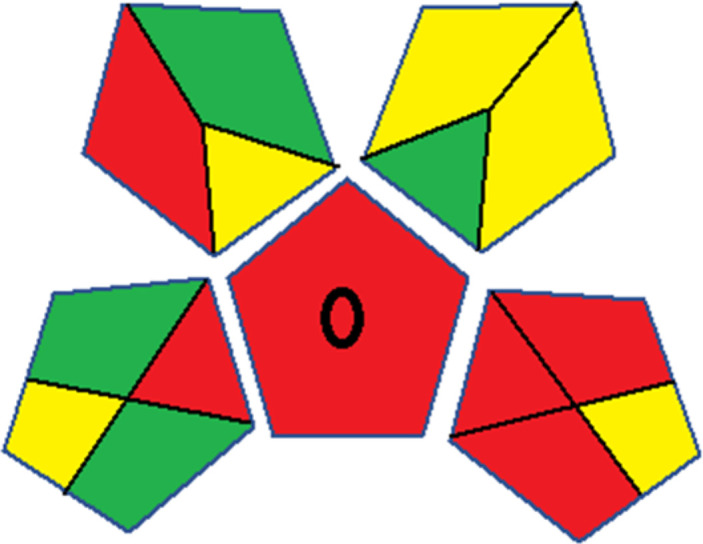	19
	Collection (1)	Offline (red)		
	Preservation (2)	None (green)		
	Transport (3)	Required (yellow)		
	Storage (4)	None (green)		
	Type of method: direct or indirect (5)	Extraction required (red)		
	Scale of extraction (6)	Micro-extraction (yellow)		
	Solvents/reagents used (7)	Non-green solvents/reagents used (red)		
	Additional treatments (8)	None (green)		
	**Reagent and solvents**			
	Amount (9)	<10 mL (green)		
	Health hazard (10)	NFPA = 2, moderate toxicity (yellow)		
	Safety hazard (11)	NFPA = 3, high flammability (yellow)		
	**Instrumentation**			
	Energy (12)	>1.5 kW h per sample (red)		
	Occupational hazard (13)	Emission of vapours to the atmosphere (red)		
	Waste (14)	1–10 mL (yellow)		
	Waste treatment (15)	No treatment (red)		

17	**Sample preparation**		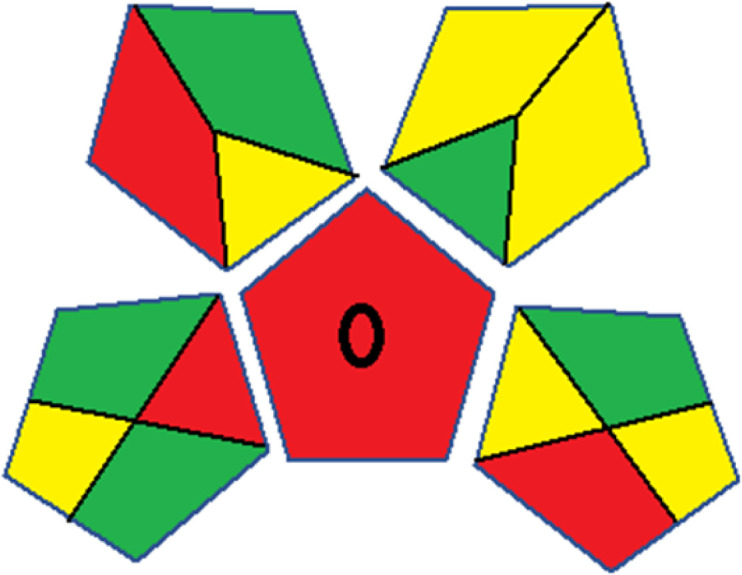	31
	Collection (1)	Offline (red)		
	Preservation (2)	None (green)		
	Transport (3)	Required (yellow)		
	Storage (4)	None (green)		
	Type of method: direct or indirect (5)	Extraction required (red)		
	Scale of extraction (6)	Micro-extraction (yellow)		
	Solvents/reagents used (7)	Non-green solvents/reagents used (red)		
	Additional treatments (8)	None (green)		
	**Reagent and solvents**			
	Amount (9)	< 10 mL (green)		
	Health hazard (10)	NFPA = 2, moderate toxicity (yellow)		
	Safety hazard (11)	NFPA = 3, high flammability (yellow)		
	**Instrumentation**			
	Energy (12)	≤1.5 kW h per sample (yellow)		
	Occupational hazard (13)	Hermetic sealing of analytical procedure (green)		
	Waste (14)	1–10 mL (yellow)		
	Waste treatment (15)	No treatment (red)		

18	**Sample preparation**		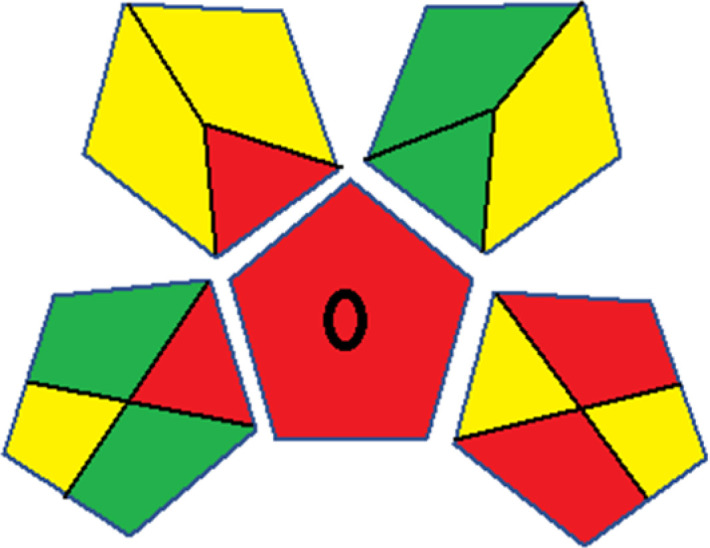	24
	Collection (1)	Offline (red)		
	Preservation (2)	None (green)		
	Transport (3)	Required (yellow)		
	Storage (4)	None (green)		
	Type of method: direct or indirect (5)	Extraction required (red)		
	Scale of extraction (6)	Macro-extraction (red)		
	Solvents/reagents used (7)	Green solvents/reagents used (yellow)		
	Additional treatments (8)	Simple treatments (yellow)		
	**Reagent and solvents**			
	Amount (9)	< 10 mL (green)		
	Health hazard (10)	NFPA = 1, slight toxicity (green)		
	Safety hazard (11)	NFPA = 3, high flammability (yellow)		
	**Instrumentation**			
	Energy (12)	≤1.5 kW h per sample (yellow)		
	Occupational hazard (13)	Emission of vapours to the atmosphere (red)		
	Waste (14)	1–10 mL (yellow)		
	Waste treatment (15)	No treatment (red)		

19	**Sample preparation**		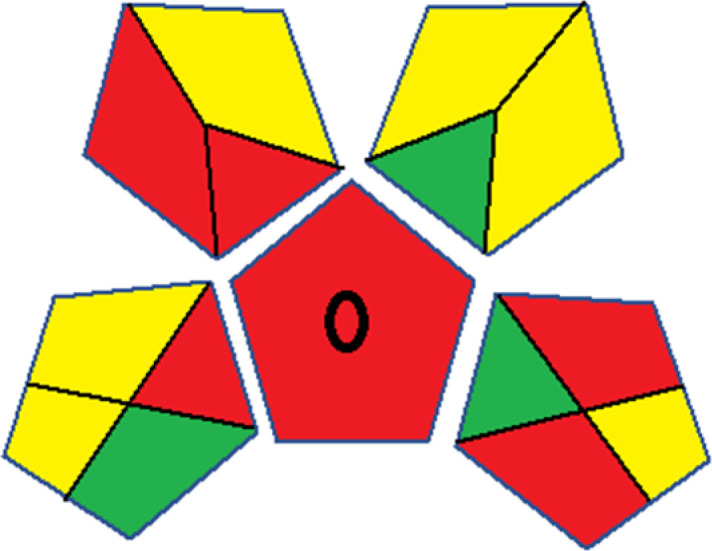	30
	Collection (1)	Offline (red)		
	Preservation (2)	None (green)		
	Transport (3)	Required (yellow)		
	Storage (4)	Under normal conditions (yellow)		
	Type of method: direct or indirect (5)	Extraction required (red)		
	Scale of extraction (6)	Macro-extraction (red)		
	Solvents/reagents used (7)	Non-green solvents/reagents used (red)		
	Additional treatments (8)	Simple treatments (yellow)		
	**Reagent and solvents**			
	Amount (9)	< 10 mL (green)		
	Health hazard (10)	NFPA = 2, moderate toxicity (yellow)		
	Safety hazard (11)	NFPA = 3, high flammability (yellow)		
	**Instrumentation**			
	Energy (12)	≤0.1 kW h per sample (green)		
	Occupational hazard (13)	Emission of vapours to the atmosphere (red)		
	Waste (14)	1–10 mL (yellow)		
	Waste treatment (15)	No treatment (red)		

Assessment of whiteness profiles was conducted using the RBG 12 algorithm. The assessment procedure involved assigning scores to all 19 methods, evaluating each attribute.^48^ The ‘red’ criteria included the following: application scope – R1 (including the number of determined analytes), lower limit of quantification (LLOQ) – R2, precision – R3, accuracy – R4. For the ‘green’ criteria, the selected aspects were toxicity of reagents measured by the number of Globally Harmonized System of Classification and Labelling of Chemicals (GHS) pictograms of the employed reagents – G1, quantity of reagents and waste produced – G2, consumption of energy – G3, direct impact on the user (safety or occupational hazards) – G4. Regarding the ‘blue’ criteria: cost efficiency – B1, time efficiency – B2, requirements including sample amount utilized for analysis and other needs such as advanced instruments and skills personnel– B3, and operational simplicity including miniaturization, integration and degree of automation – B4.

**Fig. 2:**
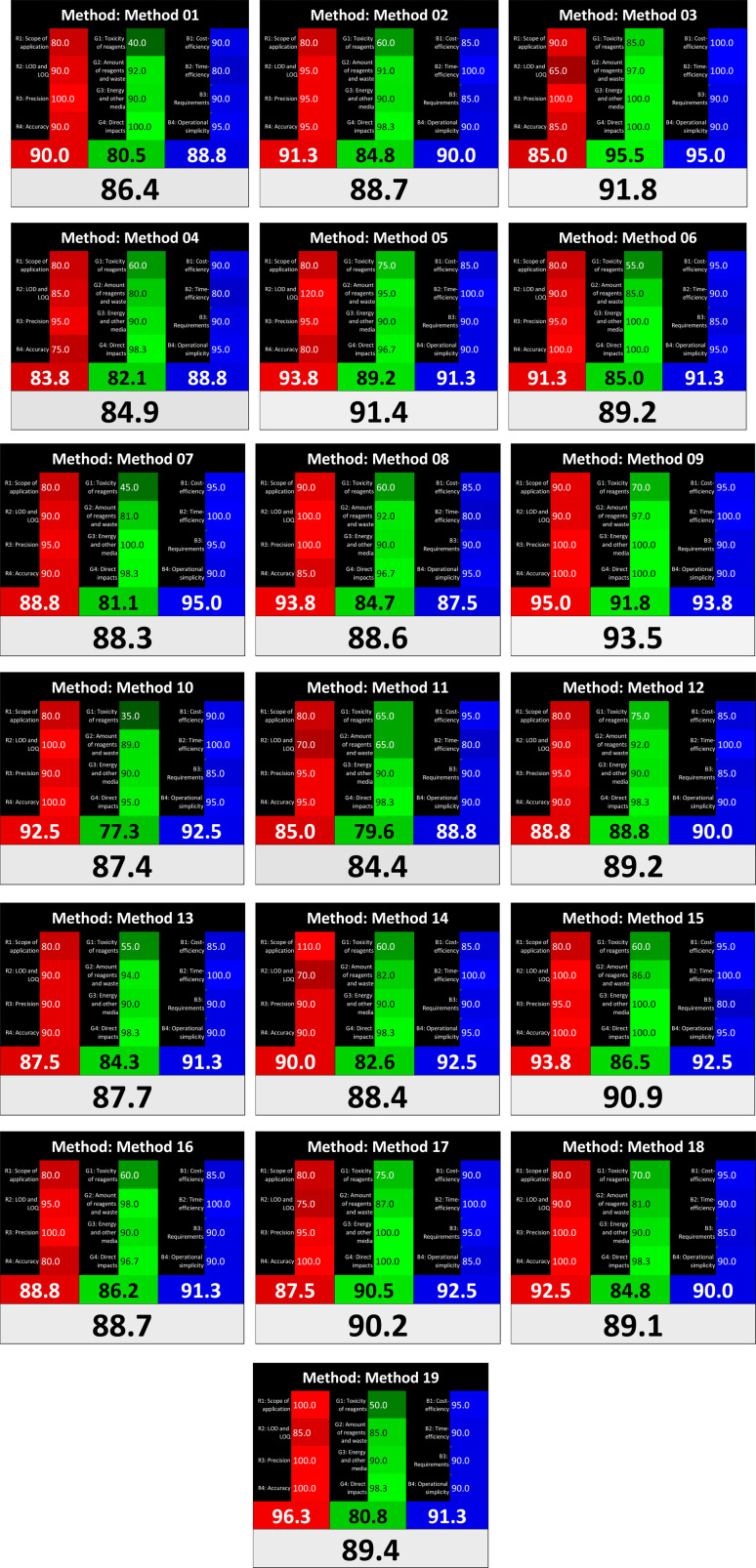
Comparison of the whiteness profiles of the 19 methods for determining favipiravir in biological fluids using the RGB 12 algorithm. Red criteria (method performance) included scope of application (R1); lower limit of quantification (R2); precision (R3) and accuracy (R4). Green criteria (method greenness) included: toxicity of reagents (G1); amounts of reagent and waste (G2); energy consumption (G3) and direct impacts (G4). Blue criteria (method effectiveness) included: cost-efficiency (B1); time-efficiency (B2); requirements: sample consumption and the need for advanced instruments and skilled personnel (B3) and operational simplicity (B4).

**Fig. 3:**
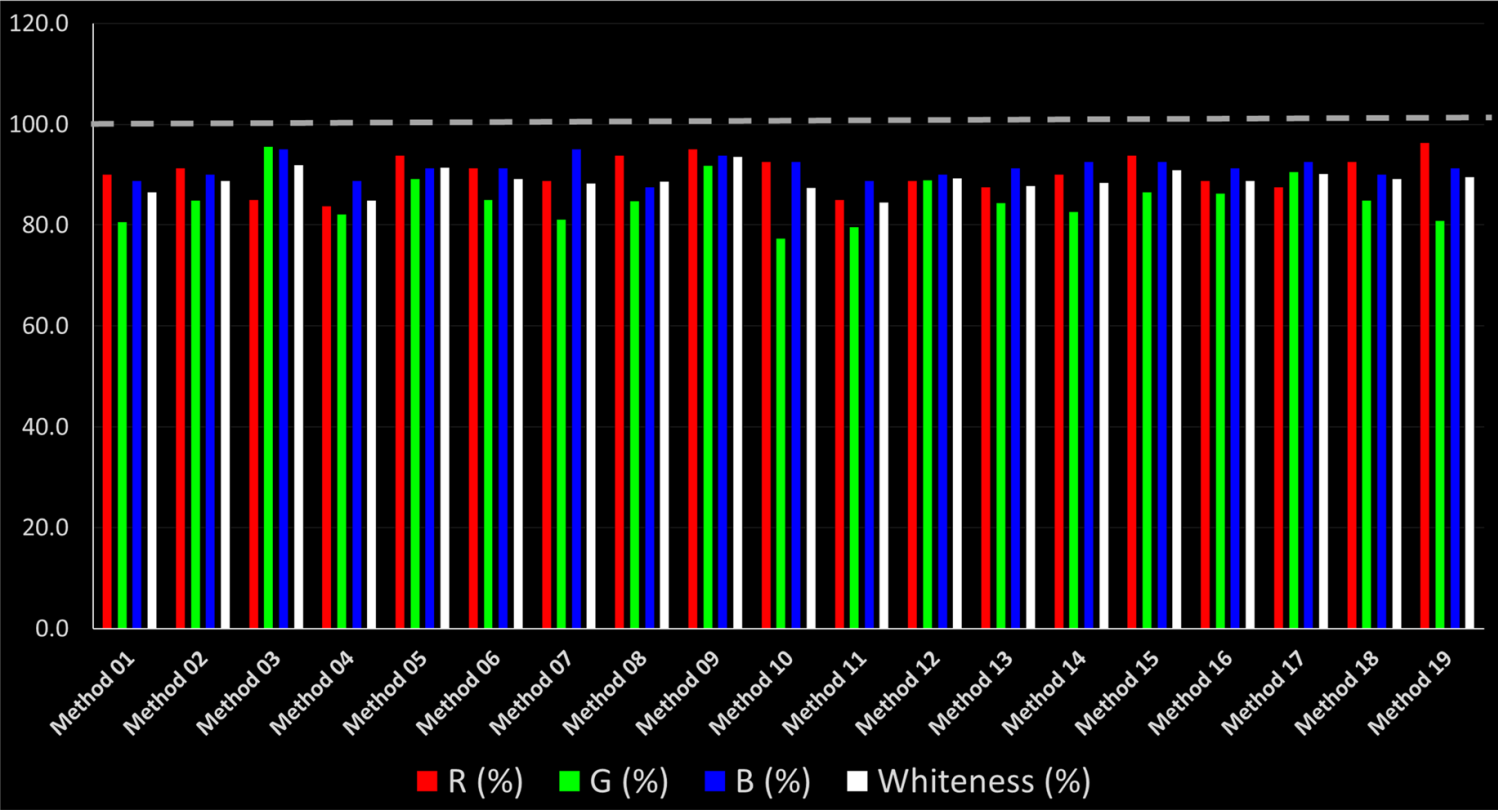
Comparison of the main assessment outcomes of the 19 methods used to determine favipiravir in biological fluids, as derived from the RGB 12 analysis. The white dotted line represents 100%, indicating complete suitability for the planned application.

As WAC concept is relatively new, there are not established and universally accepted rules for assessing these methods yet.^53^ So, scoring these criteria is mostly based on subjective judgment, although efforts are made to ensure a comprehensive assessment. In this study, efforts were made to make the assessment as fair as possible, and specific rules were set for each evaluated method to determine their scores. Although the choice of rules might have some subjectivity, it is enough to make a reasonable comparison between methods. The scoring rules used to evaluate analytical methods for favipiravir in biological matrices are detailed in file 2 of the ESI.† Calculations were conducted using an Excel spreadsheet.^48^Fig. 2 shows the assessment results of the 19 methods, illustrating the outcomes for each method, while a comparison between main assessment outcomes is illustrated in Fig. 3. All whiteness assessment calculations using the Excel spreadsheet are presented in file 3 of the ESI.†

## Results and discussion

### Assessment of methods' greenness profiles

Several comparative studies included NEMI as one of their assessment tools. However, the findings suggested that NEMI lacked the reliability needed for discriminative comparative results.^54–57^ Consequently, it was not employed in the current work. The summary of comparison results for all three greenness assessment tools is presented in Table 2. AES and GAPI provide semi-quantitative results, while AGREE and RBG 12 results are quantitative and qualitative.^43^ In addition, Table S1 in file 4 of the ESI† provides a summary of all methods ranked based on their greenness assessment. For a more thorough and comprehensive evaluation, it is advised to utilize different greenness assessment tools when analyzing the environmental impact of analytical methods. This is crucial because not all tools consider all 12 principles of GAC.^43^

The comparison of the eco-friendliness of the 19 methods using GAPI and AGREE metrics revealed that Method 3 emerged as the most eco-friendly approach for determining favipiravir in biological matrices. It exhibited the highest greenness profile as determined by both the GAPI and AGREE tools, with 6 green pictograms in GAPI and the highest AGREE score of 0.65, further substantiating its superior environmental performance. Method 12 secured the second position, displaying 6 green pictograms in GAPI and an AGREE score of 0.56. Similarly, Method 9 and Method 17 exhibited favorable environmental characteristics, each acquiring 5 green pictograms in GAPI and AGREE scores of 0.61 and 0.60, respectively.

It is important to note that the lower AGREE score of Method 12, compared to Methods 9 and 17, can be attributed to the utilization of LC-MS/MS, which enhanced the method sensitivity. This methodological choice introduced specific elements that contributed to the reduction in its overall AGREE score. This finding highlights the impact of methodological decisions on environmental assessments, prompting further consideration of the trade-offs involved in selecting sensitivity-enhancing techniques.

Conversely, Method 19 exhibited the least environmentally friendly profile among the evaluated methods, attaining only 2 green pictograms in GAPI. Additionally, it received the lowest AGREE score of 0.53, indicating a comparatively lower level of environmental sustainability. This can be attributed primarily to the use of lengthy extraction procedures involving hazardous organic solvents and extended elution times for chromatographic separation, resulting in high amounts of hazardous solvents (Table 1).

The AES tool further substantiated the superior environmental performance of Method 3 (91), emphasizing the need for a multi-faceted evaluation to achieve a comprehensive understanding of the ecological impact of analytical methods. It was followed by Method 17, with a score of 86, and Methods 9 and 12, both scoring 85. The least environmentally friendly method was Method 10, with a score of 67. These findings emphasize the complex relationship between methodological choices and environmental sustainability, guiding future considerations in method selection and development.

### Assessment of methods' whiteness profiles

While existing greenness assessment scales enable the comparison of methods based on ecological aspects,^6,37–39^ they do not consider parameters that determine the method functionality such as method effectiveness and practicality, thus they do not represent the overall quality of the method.^58^ Conversely, the WAC concept goes beyond the green metric approach by including additional criteria beyond environmental considerations.^48^ Through the application of these principles, the assessment using the WAC approach enables a more comprehensive analysis of both method performance and its practical advantages. Nonetheless, subjectivity persists. Therefore, this study sought to minimize such subjectivity by establishing scoring rules for each criterion. These rules were consistently applied to all 19 compared methods, as detailed in the ESI (File 2).†

Contrary to the greenness assessment results, according to RGB 12 analysis, Method 9 achieved the highest whiteness score (93.5%). Method 3 secured the second-highest position with a whiteness score of 91.8%, closely followed by Method 5 in third place with a whiteness score of 91.4%. Below is a detailed explanation of the whiteness assessment results for all criteria.

### Red criteria (method performance)

All 19 tested methods showed acceptable analytical performance with CS_red_ ranging between 83.8% to 96.3%. Method 19 achieved the highest analytical performance (CS_red_ = 96.3%) due to its broad scope of application, good precision (*i.e.*, <5%) and accuracy of the micellar ultra performance liquid chromatography – UV (UPLC-UV) method. Method 9, utilizing UPLC-UV, achieved second place with a CS_red_ score of 95.0%, attributed to its enhanced precision and accuracy (*i.e.*, 98–103%). Although Method 5 (LC-MS/MS) exceeded Methods 19 and 9 in sensitivity, boasting a very low LLOQ (0.78 ng mL^−1^) due to the utilization of the highly sensitive MS/MS detector, Methods 19 and 9 demonstrated superior scope of application, precision, and accuracy. Consequently, Method 5 secured third place with a CS_red_ score of 93.8%. Sharing third place with Method 5 were Method 8 (Quick, Easy, Cheap, Effective, Rugged, and Safe ‘QuEChERS’ coupled to (LC/MS/MS) and Method 15 (HPLC-UV).

Method 4 (HPLC-UV), despite exhibiting good precision (*i.e.*, < 10%), demonstrated poor accuracy (*i.e.*, 69–73%), limited scope of application, and a high LLOQ (*i.e.*, 500 ng mL^−1^), resulting in the lowest CS_red_ value among all 19 methods (*i.e.*, 83.8%).

### Green criteria (method greenness)

Unlike the red criteria, Method 3 emerged as the top performer in terms of environmental sustainability, achieving a CS_green_ of 95.5%, consistent with the results obtained from the greenness assessment tools. This success is attributed to low energy consumption, absence of occupational hazards, a relatively low number and toxicity of reagents, and minimal waste generation of the UPLC-UV method utilized. Method 9 secured second position with CS_green_ = 91.8%, followed by Method 17 with CS_green_ = 90.5%.

The lowest CS_green_ value was obtained by Method 10 (HPLC-UV), 77.3%. Although the occupational hazards were low – exposure to high temperature (during reflux) – reagents toxicity, consumption of energy and waste generation were high. This correlated with the extensive use of reagents during the preparation of the gadolinium-based magnetic ionic liquid.^13^

### Blue criteria (method effectiveness)

Methods 3 and 7 achieved the highest scores, with CS_blue_ = 95.0%. This can be attributed to their cost-effective use of the HPLC-UV technique. Method 7 employed a simple protein precipitation step, while Method 3 did not require any prior extraction step. Both methods displayed shorter total sample preparation and chromatographic separation run times (14.8 and 24 minutes for Methods 3 and 7, respectively) and utilized relatively small sample volumes (1 and 0.2 mL for Methods 3 and 7, respectively). In addition, neither method required sophisticated instruments. Method 9 (UPLC-UV) secured second place with CS_blue_ = 93.8%.

Methods 10 (HPLC-UV), 14 (2D-LC-MS/MS), 15 (HPLC-UV), and 17 (HPLC-UV) attained third place, each achieving a CS_blue_ value of 92.5%. While Methods 10, 15, and 17 employed cost-effective HPLC-UV technology, Method 14 utilized a more expensive and sophisticated MS/MS detector, requiring skilled personnel for operation. However, Method 14 demonstrated time efficiency, with a total sample preparation and analysis time of 10 minutes and employed online solid-phase extraction (SPE) sample preparation, contributing to its method score in terms of automation. As a result, all four methods tied for third place with the same CS_blue_ score. Method 8 obtained the lowest CS_blue_ score (*i.e.*, 87.5%), primarily attributed to the use of expensive LC-MS/MS equipment, which also required skilled personnel for operation, thus reducing the method score. Moreover, the method's sample preparation and analysis time was lengthy (approximately 73 min per sample), and the absence of integration further contributed to the reduction in the method's CS_blue_ score.

Overall, Method 9 exhibited the best whiteness profile results, achieving high scores for the red, green, and blue attributes. It secured second place for the red and green attributes, and also placed second after Methods 3 and 7 in the blue attributes. Method 3, the second-ranked method, outperformed Method 9 in the green and blue aspects but lagged behind in the red criteria (*i.e.*, method performance), primarily due to the high LLOQ (*i.e.*, 2000–5000 ng mL^−1^ compared to 100 ng mL^−1^ for Method 9) and lower accuracy (*i.e.*, 82–103% for Method 3 compared to 98–103% for Method 9). This resulted in an overall red score (CS_red_) of 85 and 95 for Methods 3 and 9, respectively, contributing to the overall whiteness score of 93.5% for Method 9, placing it in the first position ahead of Method 3 with 91.8%, which was ranked second in the overall whiteness score.

The data depicted in Fig. 2 and 3 illustrate the difference in ratings across all assessment criteria for individual methods, and a comparison between the main assessment outcomes of all 19 methods. In addition, Table S2 in file 4 of the ESI† presents the ranking of all 19 methods based on the red, green, blue, and overall whiteness criteria. Method 5 secured third place with a whiteness score of 91.4%, followed closely by Method 15 (90.9%). While both methods excelled in method performance (red criteria) and practical effectiveness (blue criteria), achieving scores of 91.3% and 92.5% for the blue criteria for Methods 5 and 15, respectively, as well as 93.8% for the red criteria for both methods, they showed comparatively lower performance in the green criteria, with scores of 89.2% and 86.5%, respectively. The poorest overall whiteness profile was obtained by Method 11 (84.4%), displaying poor method greenness and performance criteria with scores of 79.6% and 85.0%, respectively. This can be attributed to several factors, including the high LLOQ of the HPLC-UV method utilized (*i.e.*, 3100 ng mL^−1^), limited scope of application, toxicity, and the excessive amounts of reagents used, resulting in high waste generation.^25^

In conclusion, a comprehensive analysis of the results obtained from both the greenness and whiteness assessment for the 19 methods provided insights into their environmental and functional aspects. The greenness assessment, incorporating metrics such as GAPI and AGREE, identified Method 3 as the most eco-friendly method. However, the whiteness assessment ranked this method as the second-best, placing Method 9 in the top position. Method 9 also ranked third according to ASE, GAPI, and AGREE metric assessments. This indicates that factors such as method performance (red criteria) and practicality (blue criteria) play a significant role in the overall assessment of analytical methods.

Conversely, Method 12 exhibited an excellent ASE score of 85, securing third place among all 19 methods. However, its AGREE score of 0.56 placed it at the eleventh position. Interestingly, GAPI results highlighted Method 12 with 6 green pictograms, emphasizing its status as one of the best environmentally friendly methods among all 19 methods. In the whiteness assessment, the overall score of 89.2% positioned Method 12 at the seventh place, a result that aligns well with the AGREE evaluation. This can be explained because Method 12 utilized LC-MS/MS technique, which can enhance method performance (sensitivity and selectivity), but on the other hand, could negatively impact the method's greenness profile by increasing energy consumption. In addition, the use of expensive LC-MS/MS instrumentation would affect the method sustainability, specifically the blue score, due to the financial resources required for instrument acquisition and maintenance, as well as the need for skilled personnel for operation and data interpretation. As a result, Method 12 secured the thirteenth place in the blue criteria and the seventh place in the overall whiteness assessment due to its enhanced method performance (red criteria) attributed to the utilization of LC-MS/MS technology.

These findings support the conclusion that to develop a sustainable method for the bioanalysis of favipiravir, it is crucial to employ short sample preparation and analysis times using a cost-effective analytical technique such as LC-UV, with proper optimization of column selection, mobile phase composition, and automation. The utilization of LC-MS/MS, while enhancing sensitivity and selectivity, has drawbacks, including increased energy consumption and high costs associated with instrumentation and maintenance. However, in cases where very low limits of detection or enhanced selectivity are required, LC-MS/MS becomes indispensable despite these drawbacks. This highlights the need for a balanced approach that prioritizes sustainability whenever possible.

Finally, the summary of the above findings, collectively stresses the importance of a holistic approach in method evaluation, acknowledging the broader sustainability implications beyond just environmental considerations. The comprehensive insights provided by both greenness and whiteness assessments guide a more informed and balanced selection of analytical methods, ensuring integration of environmental responsibility, analytical performance, and practical efficiency.

### Strengths and limitations of greenness and whiteness metrics in this study

The AES metric is straightforward and relies on specific assessment criteria. It provides semi-quantitative results. It takes into account the quantities of reagents used and waste generated, making it suitable for comparative studies of different analytical methods. However, the primary limitation of the AES metric is producing a numerical value of the result without providing explanations of non-environmentally-friendly aspects within the analyzed method.^59^

The primary advantage of GAPI is its comprehensive assessment of the entire analytical processes. It considers all relevant factors including sample preparation, transportation, reagents, and analytical instruments. In addition, GAPI is highly efficient in the comparative analysis of multiple analytical methods, aiding in the selection of the most environmentally friendly option. It provides detailed information for every step of the analytical process, highlighting areas that require improvement for enhanced greenness. However, the main disadvantage of GAPI is its complexity compared to AES.^38^

AGREE's primary advantage is its ability to highlight the strengths and weaknesses across the twelve principles of GAC.^39^ In addition, its overall score provides both quantitative and qualitative information regarding GAC principles. AGREE is preferred over AES due to its consideration of factors such as sample size, sample throughput (number of samples analyzed per hour), the use of bio-based solvents, and the presence of toxic reagents to aquatic life. Moreover, AGREE is favored over GAPI for its simplicity, automation, and quantitative results, enabling comparable conclusions regarding the ecological impact of methods with reduced effort.

Overall, it is recommended to use more than one greenness assessment tool to obtain a comprehensive assessment of a method's greenness profile. However, even when combined, these metrics still lack consideration for essential sustainability aspects such as method performance and cost-effectiveness.

On the other hand, whiteness assessment, utilizing the WAC concept (RGB12 metric), offers a more comprehensive view by considering not only ecological aspects but also analytical and practical parameters. However, it is noteworthy that while the whiteness assessment using RGB12 tool provides the most comprehensive evaluation, incorporating method performance and effectiveness in addition to the method's green aspects, its main limitation lies in subjectivity. This subjectivity can arise from the interpretation of scoring criteria and the weighting of different metrics, which may vary among evaluators. To address this limitation, establishing well-defined scoring criteria and guidelines for interpretation could enhance objectivity and consistency in whiteness assessment. One solution could involve the development of automated tools for calculations, which would reduce the potential for human bias and facilitate standardized scoring across different evaluations. These automated tools could incorporate predefined scoring algorithms based on agreed-upon criteria, ensuring uniformity in assessment outcomes. In addition, providing clear explanations and examples for each scoring criterion could help mitigate subjectivity by ensuring evaluators have a common understanding of how to apply the criteria in practice. By addressing the subjectivity issue and enhancing the objectivity of whiteness assessment using the RGB12 tool, researchers and laboratories can more confidently utilize this comprehensive evaluation tool to inform method selection and promote the development of sustainable and efficient analytical practices.

## Conclusion and future perspective

In summary, the comprehensive evaluation of 19 liquid chromatographic methods for the bioanalysis of favipiravir has provided valuable insights into their environmental impact, analytical performance, and practical efficiency. By utilizing both green and white analytical assessment tools, the study assessed the environmental impact and overall sustainability of each method.

Greenness assessment tools indicated that Method 3 was the most eco-friendly among the 19 methods, while the whiteness assessment revealed that Method 9 performed the best due to its superior method performance, time and cost efficiency, and good greenness profile. This highlights the crucial link between environmental responsibility and method quality, emphasizing the importance of considering broader sustainability aspects.

The AES metric provides a straightforward, semi-quantitative assessment of analytical methods based on reagent usage and waste generation, but it lacks detailed explanations of non-eco-friendly aspects. GAPI offers a comprehensive analysis of entire processes, while AGREE highlights strengths and weaknesses across GAC principles and provides both quantitative and qualitative information. Whiteness assessment using the RGB12 metric is comprehensive but subjective, necessitating well-defined criteria and automated tools for enhanced objectivity and consistency.

Developing sustainable and efficient analytical methods for favipiravir bioanalysis can be enhanced by exploring alternative greener solvents, such as natural deep eutectic solvents or those derived from renewable resources, to replace conventional organic solvents in sample preparation. This reduces environmental impact and health risks. In addition, utilizing miniaturized and automated techniques, such as chip-based separation systems, can minimize solvent and sample consumption, reduce waste, and enhance throughput. Methods that support *in situ* analysis should also be considered. Integrating green sample preparation methods, like solid-phase microextraction (SPME) and dispersive liquid–liquid microextraction (DLLME), can further enhance the process and reduce solvent use. Furthermore, optimizing chromatographic conditions, including column selection and mobile phase composition, can improve separation efficiency, reduce analysis time, and minimize solvent consumption.

## Data availability

The data supporting this article have been included as part of the ESI.†

## Conflicts of interest

There are no conflicts of interest to declare.

## Supplementary Material

RA-014-D4RA03017F-s001
